# Recent Studies on Berry Bioactives and Their Health-Promoting Roles

**DOI:** 10.3390/molecules27010108

**Published:** 2021-12-24

**Authors:** Beyza Vahapoglu, Ezgi Erskine, Busra Gultekin Subasi, Esra Capanoglu

**Affiliations:** 1 Department of Food Engineering, Faculty of Chemical and Metallurgical Engineering, Istanbul Technical University, Maslak, Istanbul 34469, Turkey; beyzavahapoglu@gmail.com (B.V.); sendile@itu.edu.tr (E.E.); bgultekin@cumhuriyet.edu.tr (B.G.S.); 2 Hafik Kamer Ornek Vocational School, Cumhuriyet University, Sivas 58140, Turkey

**Keywords:** berry fruits, phenolic compounds, bioactive compounds, healthy diet

## Abstract

Along with the increased knowledge about the positive health effects of food bioactives, the eating habits of many individuals have changed to obtain higher nutritional benefits from foods. Fruits are among the most preferred food materials in this regard. In particular, berry fruits are important sources in the diet in terms of their high nutritional content including vitamins, minerals, and phenolic compounds. Berry fruits have remedial effects on several diseases and these health-promoting impacts are associated with their phenolic compounds which may vary depending on the type and variety of the fruit coupled with other factors including climate, agricultural conditions, etc. Most of the berries have outstanding beneficial roles in many body systems of humans such as gastrointestinal, cardiovascular, immune, and nervous systems. Furthermore, they are effective on some metabolic disorders and several types of cancer. In this review, the health-promoting effects of bioactive compounds in berry fruits are presented and the most recent in vivo, in vitro, and clinical studies are discussed from a food science and nutrition point of view.

## 1. Introduction

The number of diet-related diseases, such as diabetes and obesity, as well as cardiovascular, immune-related, and nervous system disorders are increasing day by day and are gradually becoming a global health problem [1]. However, the extent of knowledge regarding the health effects of food ingredients such as vitamins, minerals, and some bioactive compounds is also increasing concomitantly with the interest and awareness of consumers. Based on this consciousness, individuals reform their eating habits predominantly in accordance with balanced nutrition and wellbeing that could be obtained from foods [1]. Consequently, fruits have become an important component of diets with their high fiber, vitamin, mineral, and phenolic contents.

Berry fruits with their sweet taste, unique aroma, and high phenolic contents are important sources contributing to the improvement of diet quality. The most commonly consumed berry varieties are blueberries, strawberries, black and red raspberries, cranberries, black currants, lingonberries, chokeberries, elderberries, and blackberries [2]. Depending on the species, they usually have a color range between red to purple or black. In addition to being consumed fresh or dried, berries are also utilized as different forms of food products such as jams, jellies, and drinks. Environmental conditions during cultivation are critical factors that affect fruit components and therefore the final quality so that many of them have their unique regions under optimum cultivation conditions [2]. What all variants have in common is that they contain high levels of phenolic compounds, including flavonoids, phenolic acids, tannins, stilbenes, and lignans [3].

There has been a significant rise in studies related to the health benefits of berries. Anti-diabetic, anti-obesity [4], anticarcinogenic [5,6], and anti-inflammatory [7] activities of berries have been reviewed broadly in many recent studies. In addition, the Berry Health Benefits Symposium has been held biennially since 2005 to discuss the health promoting effects of berries, including their potential impacts on cardiovascular systems and gut health [8]. The present study aimed to provide an overview of the promising health benefits of berries by focusing on the most important diseases with respect to global health problems from a food science and nutrition perspective. The effects of berries in relation to some particular disorders were discussed. The action mechanisms of berries on different diseases and their interrelation with pathogeny were also briefly summarized indicating their importance for being used/consumed routinely in the daily diet of individuals.

## 2. Nutritional and Bioactive Values of Berries

It is quite widely believed that fruits are of great importance in the human diet, providing numerous health benefits. With the advancement and increase in research, scientists are able to further delve into the details and reasons for these advantages. Berries are noteworthy fruits in this sense, providing both improvement in health as well as their physical allure and palatability. Multiple types of berries exist in nature, both edible and inedible, in varying colors, shapes, and tastes. The composition of berries, and consequently their nutritional advantages, depends on a multitude of factors such as breed, soil type, climate, location, time of harvest, handling, and storage conditions [9]. With that said, in general, berries contain both micro and macronutrients, are high in dietary fiber and fructose, and contain important vitamins, minerals, and fatty acids. The main vitamins present in berries are vitamins A, C, and E, and the B complex vitamins, contributing to their overall antioxidant capacity [10].

Berries are fairly rich in polyphenols, which are secondary metabolites of various fruits, vegetables, and cereals, and over 8000 types of phenolic compounds have been found. They are generally classified in the following categories in terms of their chemical structures: phenolic acids, flavonoids, lignans, anthocyanins, and stilbenes [11]. In general, the abovementioned phenolic compounds, along with the vitamins and minerals, make berries an important source of bioactive compounds (BAC), leading them to have antioxidant, antimicrobial, antifungal, chelating, and pigmentation properties [10]. Due to these health benefits of the BAC held in berries, they are known to have positive effects on the gastrointestinal system, metabolism-related diseases, the cardiovascular system, the immune system, the nervous system and numerous types of cancers [12]. Table 1 shows commonly studied berries and their phenolic contents extracted from various studies. These values can differ in literature as the source and type of berry, assay method, as well as other affecting factors listed above, can have an impact on their phenolic values.

Owing to their favorable taste, berries are mostly consumed in their fresh form without the need for processing or altering the taste. This is an incredible advantage in the sense that the BAC they contain is at its peak level and is therefore of great benefit to consumers [9]. Berries have both different types and amounts of phenolic compounds, leading to divergence in colors and antioxidant capacities. There are numerous studies which look into the phenolic contents and antioxidant capacities of berries utilizing a variety of methods such as 2,2-diphenyl-1-picrylhydrazyl (DPPH), 2,2′-azino-bis-3-ethylbenzotiazolin-6-sulfonic acid (ABTS), ferric reducing antioxidant power (FRAP), and cupric ion reducing antioxidant capacity (CUPRAC) assays [34]. Total phenolic content results can be expressed in gallic acid (GAE) or quercetin (QE) equivalents, while their antioxidant capacities are usually expressed in Trolox (TE) or ascorbic acid equivalents (AAE). These variations of the units often make it quite challenging for comparisons of different studies despite all representing antioxidant capacity [35].

A comparative study of six berries, honeyberry, blueberry, mandarin melon berry, mulberry, chokeberry, and Korean black raspberry, was conducted to research their individual characteristics and how their nutritive values are affected by composition [22]. Antioxidant activity was evaluated with a combination of three tests consisting of ABTS, DPPH, and FRAP and the results showed that chokeberry exhibited the highest antioxidant activity, closely followed by Korean black raspberry, while the melon berry had the lowest antioxidant activity. The research showed a positive correlation between the amounts of flavonoids, phenolics, and anthocyanins present in the berries and their antioxidant activities. The total phenolic contents of chokeberry, Korean black raspberry, and melon berry were measured to be 194.61, 144.71, and 31.16 GEC µg/mL, respectively. These were parallel to their respective antioxidant activities where the values of chokeberry and Korean black raspberry were found to be 10 times higher than that of melon berry. Another detailed study focusing on berries covered their total anthocyanin, total flavonoid, and DPPH radical scavenging activity amongst other antioxidant capacity assays, using both spectrophotometric and colorimetric methods [36]. Raspberries, black currants, red currants, white currants, white gooseberries, red gooseberries, blackberry, goji, and three types of blueberries were evaluated. The highest total anthocyanin (mg cyanidin-3-glucoside eq./g) and flavonoid (mg rutin eq./g) contents were found in blackcurrants with the results being 7.59 mg/g and 24.78 mg/g, respectively. Blackcurrants were also seen to have the most promising antioxidant capacity while goji berries had the lowest among all. Trolox equivalent antioxidant capacity (TEAC; mmol Trolox eq./g), FRAP (mmol Fe^2+^/g), and radical DPPH of blackcurrant extract were 12.09 mmol/g, 10.29 mmol/g, and 0.20 mg/L, respectively. For goji berry extracts, these results were 2.79 mmol/g, 1.46 mmol/g, and 1.18 mg/L, respectively. The values of the remaining berries were spread out between these high and low points, further showing the divergences of berries in terms of their constituents.

A different study determined the types and measured the amounts of the phenolic compounds in fruits, particularly those available as commercial juices in the United Kingdom [27]. Amongst the various types of fruits assessed, cranberries and grapes were also studied. Their results showed that purple grape juice was the lead in terms of total phenolic content, while white grape juice was the lowest, with the measured results being 7.5 mmol GAE/L and 0.9 mmol GAE/L, respectively. The situation was observed for antioxidant activity as well, with cranberry juice also being noted for its high antioxidant activity. Okatan conducted a comparative study evaluating the total phenolic and anthocyanin contents along with the antioxidant activity of six different berries: redcurrant, blackcurrant, red raspberry, blackberry, gooseberry, and jostaberry [13]. Blackberry was found to have the highest antioxidant and anthocyanin levels, with values of 426.26 mg TE/100 g FW and 226.33 mg/100 g FW, respectively. Jostaberry had the highest value of total phenolic contents as 1593.92 mg GAE/100 g FW. Redcurrant generally had the lowest values out of all six berries with its total phenolics, antioxidant, and anthocyanin values being 8.45 mg GAE/100 g FW, 24.41 mg/100 g FW, and 8.70 mg/100 g FW, respectively.

Briefly, it could be deduced that berries are quite beneficial to human health in terms of their abundant amount of total phenolic acids, anthocyanins, flavonoids, and thus high antioxidant capacities. Studies generally showed that polyphenols in particular were highly linked with a remarkable level of antioxidant capacity which demonstrates the ability to inhibit reactive oxygen species (ROS). Due to this reason, berry polyphenols have reputable potential to demote adverse health effects of ROS-induced diseases and disorders [37,38]. It should be noted that different amounts and types of phenolic compounds were determined in varying studies outlined above, even for the same berry fruit, emphasizing their critical dependency on external, agricultural, and cultivation factors.

## 3. Health Effects of Berries

### 3.1. Gastrointestinal System

Even though the effects of berries on many health issues had been investigated widely, their effects on gastrointestinal (GI) systems and disorders were comparatively less studied [39]. The gastrointestinal, or digestive, track system, which is mainly composed of the mouth, esophagus, stomach and intestines, has been examined for the effect of berry bioactives with varying numbers of in vivo and in vitro studies through increasing popularity [39,40]. Particularly, the ROS within the GI tract is the major inducer of oxidative tissue damage, sourcing from reducing the endogenous antioxidative enzymes, hence inducing oxidative stress to trigger various GI disorders such as gastritis, peptic ulcer, and inflammatory bowel disease [41,42]. Different berry bioactives in terms of varying molecular mechanism of actions have been investigated, with the nuclear factor-kappaBp65 (NF-ϰB) pathway being the most common one for many berry bioactives to be modulated [42]. Protective roles of berry bioactives against GI oxidative stress and inflammation as gastric and intestinal effects were reviewed comprehensively for each individual berry [42]. On the other hand, the effect of berry bioactives should ideally start to be examined from the mouth, where the first encounter of berries and the body takes place. The promising health effects of berry bioactives on the GI system were investigated throughout numerous studies with the most recent ones being covered in the part below.

The effect of cranberry bioactives on cariogenic virulence factors such as bacterial acidogenicity, aciduricity, and glucan synthesis was widely reviewed and their spectacular potential for cariostatic effects was emphasized, with their majority being in vitro studies [43]. Similarly, a clinical trial was conducted to examine the potential antimicrobial and anti-inflammatory effects of fermented lingonberry mouthwash in the oral cavity. The viable counts of *Streptococcus mutans* and *Candida* and *Lactobacilli* were found to be decreased, in addition to reduced visible plaque index and bleeding upon probing, with a usage of 20 mL twice daily for one week [44]. Highbush blueberry proanthocyanidins were tested for their alleviatory potential for *Porphyromonas gingivalis*-induced detrimental activities on oral mucosal cells. The outcomes showed that the integrity of the gingival keratinocyte barrier was protected, translocation and proteinase activities of *P. gingivalis* were hindered, and IL-6 and IL-8 secretions were diminished [45]. With a wider prospect, varying types of berry juices (blackcurrant, redcurrant, cranberry, and raspberry) were investigated on the most common oral pathogenic bacteria. Significant suppression effects of berry juices on targeted pathogenic bacteria coupled with minimum cytotoxic effects were observed for different applications [46].

The esophagus as another element of the GI system was examined against the bioactives of berries, but not too extensively. Ethanol extracted anthocyanin enriched fraction of black raspberry was applied in vitro on actual human microvascular endothelial cells of the esophagus. An anti-inflammatory effect was observed following the TNF-α/IL-1β activation when exposed to 100 μg/mL extract [47]. In another study, myrtle berry seed extract was used for its ameliorative and antioxidant effect on reflux-induced esophagitis in rats as well as in vitro. Significant antioxidative activity of myrtle berry seed extract was observed in vitro and a protective effect against histopathological and macroscopic changes coupled with avoiding intracellular mediator deregulation was obtained in vivo [48,49].

Positive effects of berry bioactives, specifically polyphenolics, on the protection of gastric mucosa, including ulcerative colitis, antiulcer activity, alleviation effect on morphological and histopathological damaged lesions, decreasing oxidative stress, improving antioxidative efficiency, and restoring calcium homeostasis, were commonly observed for GI tract disorders [50]. The effect of myrtle berries on GI tract system health disorders was extensively reviewed in a few studies, both in vivo and in vitro, which indicate the potential of myrtle berries to treat or avoid GI disorders such as gastroduodenal ulcer, diarrhea, and inflammatory bowel disease (ulcerative colitis) [48,51]. Ellagitannin-enriched extracts from blackberry and raspberry were applied on gastric inflammation and both extracts were observed to decrease the ulcer index of ethanol-induced oxidative stress significantly (88 and 75%, respectively) in vivo. A similar observation was also indicated from in vitro studies concerning TNF-α (NF-ϰB), IL-8 secretion, and enzymatic activity reductions [52]. Ethanol-induced peptic ulcer in rats was exposed with myrtle berry extract in a dose-dependent manner (observed gradually increased activities by 25, 50, and 100 mg/kg p.o.) and it was found that regulated enzyme metabolism induces protection against gastric and duodenal injuries [49]. These regulations were associated with the preservation of normal antioxidant enzyme activities while inhibiting the secretion abnormalities by myrtle berry extract. Similarly, *Schisandra chinensis* berry extract was used against ethanol induced gastric ulcers in mice. Significant decrease in ulcer index, malondialdehyde, TNF-α/IL-1β secretion as well as protection against mucosa lesions and increase of superoxide dismutase levels were observed [53]. Castor oil-induced diarrhea was treated in rats by feeding them with myrtle berry juice (5–10 mL/kg) in a comparative study with well-known anti-diarrhea drugs. In a dose-dependent manner, myrtle berry juice hindered intestinal motility and gastric emptying rates [54].

The gastroprotective effects of the black mulberry, raspberry, and acai berry extracts were evaluated in terms of their antioxidant and enzyme activities both in vitro and in vivo. Their ability to reduce the ulcerative lesion (83–48% with a dose range of 30–300 mg/kg) was confirmed with gastric mucosa enzyme activity and decreased inflammation [55,56]. Another gastroprotective study of black chokeberry hydro-alcoholic extract on ethanol-induced gastric ulcer in rats was evaluated and significant inhibition was observed on gastric injury formation by decreasing malondialdehyde, myeloperoxidase, and increasing superoxide dismutase, catalase, glutathione peroxidase, and interleukin (IL)-4 [57,58]. The synergistic effect of black chokeberry with *Alchemilla vulgaris* extracts was examined for indomethacin-induced gastric ulcer in rats and it was indicated that pretreatment with a combination of these extracts had significant protection and reducing effect against gastric ulcers [59]. The same berry was also tested for its amelioration potential against trinitrobenzensulfonic acid-induced colitis (inflammatory bowel disease) in a drug comparative study with sulfasalazine. It was pointed out that the berry extract had higher protective activity than sulfasalazine on macroscopic and microscopic lesions for colitis, possibly as a result of the antioxidative and anti-inflammatory effects it had [60]. In another study, the potential healing effect of the Chinese goji berry extract on aspirin-induced gastric ulcer in rats was investigated. Based on reduced lipid peroxidase malondialdehyde levels by 78%, the berry extract was confirmed to have the potential to heal in vivo gastric ulcers [61]. From a different point of view, the gastric damage of maqui berry extract, as a side effect potential, was investigated when applied with the purpose of its antinociceptive effect in mice. No signs of damage or lesion occurrence were detected during or post the experiment period [62].

Regulatory and modulatory effects of berry polyphenols on the human gut microbiota are considered to have a probiotic-like effect. Hence, it can be deduced that they diminish the symptoms of gut inflammation through the modulation of pro-inflammation cytokines and accordingly the important protective role of each individual berry on microbiota health was reviewed in detail by Lavefve et al. [63]. Encapsulated goji berry extracts were supplied as prebiotics into the growth media of probiotic bacteria naturally grown in gastric and intestinal juices containing *Bifidobacterium* and *Lactobacillus* strains. Powdered goji berry extracts stimulated the growth of targeted bacteria based on colony counts [64].

### 3.2. Metabolism-Related Diseases

Metabolism is a term that comprises all of the chemical reactions performed in a cell or organism to maintain vital functions. A defect in metabolism can cause metabolic diseases, which could be related to unhealthy eating habits. Foods taken into the body can be catalyzed to produce energy or stored in body tissues to be used when needed. When food intake becomes more than needed, the body stores most of the foods that are excessively consumed. Consequently, some diseases such as lipoidosis, obesity, and diabetes can occur in the body. In addition to nutrition, genetic defects that affect the metabolism may also cause metabolic diseases [65].

#### 3.2.1. Diabetes

Diabetes, one of the most widely seen metabolic diseases in the world, is a disease that occurs in case of dysfunction of blood sugar regulation within the body. There are two main types of diabetes including type 1 and 2. Type 1 diabetes is not preventable as its exact causes are still unclear [66]. On the other hand, type 2 diabetes is relatively preventable as it mostly occurs as a consequence of an unhealthy lifestyle such as improper diet, stress, and lack of physical activity [66]. There are different mechanisms behind type 2 diabetes, for example, patients may not be able to produce enough insulin or not be able to utilize the existing insulin. Therefore, various drugs with different action mechanisms are used in type 2 diabetes such as promoting insulin production or inhibiting sugar catalysis [67]. In addition to drug treatment, some foods can be consumed as a supplement or for the prevention of diabetes. Food supplement is especially important for people with prediabetes, those who have high blood sugar but not enough to be diagnosed as diabetic [68].

Phenolic compounds have various effects on different mechanisms involved in the development of diabetes [69]. Therefore, phenolic rich fruits such as berries have a high potential for the prevention and management of diabetes. For instance, berries have an effect on glucose intake and pancreatic β cells, which are responsible for insulin secretion through their active constituents. [70]. Therefore, the consumption of berries may exhibit a similar effect to that of drugs. Jeon et al. investigated the consumption of ethanolic extract of Aronia berry in diabetic mice. They induced type 1 diabetes using streptozotocin (100 mg/kg daily), a drug used for damaging pancreatic β cells. They observed that the daily consumption of 100 mg/kg Aronia berry extract with streptozotocin for 6 weeks almost completely inhibited the increase of blood sugar in mice. Moreover, Aronia berry extract exhibited a protective effect in pancreatic β cells [71]. In another study, Kinasih et al. studied golden berry juice consumption on blood glucose level, insulin level, and insulin resistance in rats [72]. They observed that consumption of golden berry juice (1 and 5 mL/200 g body weight/day) decreased blood glucose and insulin resistance up to 59% and 49%, respectively, and increased insulin levels up to 18% when compared to the control group (diabetic but no juice consumption). In some other studies, a decrease in glucose level and improvement in pancreatic β cells were observed with consumption of blue honeyberry [73], *Solanum nigrum* Linn berry (Umamageswari), and Canadian blueberry [74].

In clinical trials, it was observed that consumption of some berries had antidiabetic activity. For instance, the addition of wild blueberry drink to the diet exhibited an improving effect on glucose and insulin response to meals in healthy adults [75]. Similarly, consumption of freeze-dried black raspberries improved the glycemic response in young adults [76]. In addition to healthy individuals, strawberry and cranberry polyphenol juice improved insulin sensitivity in insulin-resistant, overweight, and obese people [77]. The antidiabetic effect of berries can be related to oxidative stress. Glucose-induced oxidative stress decreases insulin secretion by decreasing cell proliferation and increasing apoptosis. However, in some studies, despite the antidiabetic effect of berries, no difference in oxidative stress response was observed [76,77].

Another antidiabetic action mechanism is the inhibition of digestive enzymes. α-amylase and α-glucosidase are responsible for the catalysis of carbohydrates. Inhibition of these enzymes can delay the increase of glucose levels in serum. It is known that berries such as bilberry [78], rowanberry [79], *Lonicera caerulea* berry [80], *Vaccinium* spp. berry [81], and Andean elderberries [82] have an inhibitory activity on carbohydrate catalysis enzymes. The mechanism behind this activity depends on protein-phenolic interactions. Phenolic compounds in berries bind carbohydrate catalysis enzymes in a similar or same manner as inhibitors (e.g., acarbose). Thus, carbohydrates cannot interact with catalysis enzymes. For instance, delphinidin 3-O-galactoside, delphinidin 3-O-arabinoside, and cyaniding 3-O-glucoside in bilberry extract bound α-amylase from one side in the same way as acarbose. Owing to this property, berries play an important role in regulating blood sugar in both diabetics and healthy individuals.

#### 3.2.2. Obesity

Obesity is a disease that mainly occurs due to an unhealthy lifestyle, environmental, economic, psychological, physiologic, or genetic reasons [83]. Regulating carbohydrate and lipid metabolism and exercising are some of the most important prevention and management methods against obesity. Furthermore, the biological activities of berry bioactives against digestive enzyme inhibitors have an important role when it comes to dealing with obesity [84]. For instance, Peng et al. indicated that consumption of mulberry water extract totally inhibited the increase of body weight in hamsters after consuming a high fat diet with 2% (*w/w*) extract for 12 weeks [85]. This decrease was related to the increase of lipolysis factors (hepatic peroxisome proliferator-activated receptor R and carnitine palmitoyltransferase-1) and decrease of lipogenesis factors (fatty acid synthase and 3-hydroxy-3-methylglutaryl-coenzyme A reductase). Increase of lipolysis leads to the hydrolysis of stored fats and thus declines the fat storage in the body. On the other hand, decrease in lipogenesis prevents the increase of fat storage in the body [86]. Similarly, the effect of berry consumption on lipid storage mechanism was evaluated and significantly lower rate of lipid accumulation was found. For example, in a study performed by Wu et al., mice were fed with an ad libitum amount of blueberry and mulberry juice along with both a high-fat and low-fat diet for different groups for 12 weeks. The study indicated that blueberry and mulberry juice consumption decreased serum cholesterol levels and lipid accumulation. In addition, an improvement was observed for insulin resistance. Thus, 7.3 and 9.81% decrease in body weight was observed with bilberry and mulberry consumption, respectively [87]. Similarly, some researchers observed the positive effect of berry consumption on avoiding obesity through the decrease of lipid accumulation, serum cholesterol, and insulin resistance for blackberry [88,89], strawberry [89], blueberry [90], and cranberry [90,91,92]. Furthermore, consumption of these berries not only caused a decrease in weight gain but also increased the efficiency of metformin [93]. Metformin is a drug used for regulating glucose and lipid levels by inhibiting hepatic gluconeogenesis. When metformin and ginseng berry extract were consumed individually by high-fat-induced mice, they caused a decrease in weight gain. However, consuming ginseng berry and metformin together provided better prevention of obesity.

### 3.3. Cardiovascular System

Cardiovascular diseases are the leading cause of death worldwide [94]. An unhealthy diet, lack of physical activity, smoking, obesity, diabetes, dyslipidemia (abnormal blood lipids), and hypertension are the main reasons for cardiovascular diseases [95]. Cardiovascular risk is mostly determined by cholesterol and hypertension values. Therefore, the prevention of hypertension and high cholesterol plays an important role in reducing cardiovascular risks. Berries with their high phenolic content have the potential to prevent cardiovascular diseases by lowering those risk factors. For example, in a meta-analysis on chokeberry, it was observed that literature data show consumption of chokeberry has a significant effect on reducing cardiovascular risk factors such as cholesterol and blood pressure [96].

#### 3.3.1. Cholesterol

Cholesterol is a fat-like substance that has two types, low-density lipoprotein (LDL) and high-density lipoprotein (HDL). Both of them have different effects on the body. For example, an increase in LDL cholesterol in the blood causes a buildup of fat in the veins (plaque) [97]. Thus, vascular occlusion occurs, which results in heart attack and stroke. On the other hand, an increase in HDL cholesterol decreases the cholesterol in the blood by carrying cholesterol to the liver [97]. Berry consumption affects lipid and cholesterol metabolism and thereby decreases LDL and increases HDL cholesterol. Lui et al. reported that feeding rats with a high fat and high cholesterol diet and wild *Lonicera caerulea* berry (75, 150, or 300 mg/kg body weight per day) for 12 weeks decreased total and LDL cholesterol while increasing HDL cholesterol in serum and liver up to 47% and 36%, respectively [98]. The change of serum and liver cholesterols in the study was correlated with berry consumption amount in the diet. Moreover, the cholesterol lowering effect of berry supplementation was on the gene expression, which was related to inhibition of cholesterol absorption and lipid accumulation [98,99]. However, the mechanism of berries may differ depending on berry variety. For example, when *Lonicera caerulea* berry suppressed the mRNA and protein expression of 3-hydroxyl-3-methylglutaryl coenzyme A reductase (HMGCR), which is an important enzyme for cholesterol biosynthesis, chokeberry did not affect the expression of HMGCR in mice [98,100].

Similar results of decreasing cholesterol levels were observed for berries in clinical studies. For example, in a study, healthy but overweight adults consumed 100 g of bilberry or lingonberry with a high-fat breakfast: 4 h after breakfast, 6% and 32% decrease in total cholesterol, 7% and 24% decrease in LDL, and 13 and 23% decrease in HDL cholesterol were observed for bilberry and lingonberry, respectively, when compared with a high-fat diet which did not include fruit [101]. In a randomized cross-over study, 40 healthy non-smoker volunteers (with an average age of 63 ± 0.9) consumed berry beverages containing blueberry, blackcurrant, elderberry, lingonberry, strawberry, and tomatoes or a control beverage daily for 5 weeks. While control beverage consumption resulted in a slight increase in total and LDL cholesterols, consumption of berry beverages decreased total and LDL cholesterols in a ratio of 3.4 and 4.6%, respectively [102]. In another study, the effect of berry consumption on cardiovascular risk factors in healthy but former smoker participants was investigated [103]. In addition to a high-fat diet, it is known that smoking also increases cardiovascular risks [94]. Therefore, the risk factor reducing the effects of berries is more significant for smokers than that of nonsmokers. Participants were separated into two groups as placebo and Aronia. The Aronia group consumed 500 mg of Aronia berry extract daily. When cholesterol levels of the participants were measured after 12 weeks, total and LDL cholesterol of the Aronia group was seen to decrease by 8 and 11%, respectively. No significant difference was observed for the placebo group [103].

Reducing cardiovascular risk factors is the key to preventing cardiovascular diseases [94]. Studies so far have observed that berry fruit, beverage, or extract consumption significantly reduces cardiovascular risk factors in healthy adults. However, reducing these factors is also important for patients with cardiovascular diseases. In a study performed by Naruszewicz, patients who survived heart attacks were divided into two groups. While one group consumed 3 × 85 mg/day of flavonoid-rich extract of chokeberry combined with statin (cholesterol-lowering drug), another group consumed maltodextrin capsules instead of fruit extract. After 6 weeks, it was observed that chokeberry extract supplementation did not significantly affect cholesterol levels in patients. However, the authors mentioned that fruit supplementation can be used for the prevention of ischemic heart disease due to its reducing effect on inflammation [104]. In a similar study, patients with mildly elevated blood pressure consumed chokeberry juice and powder for 8 weeks. Similar to the previous study, no significant change with berry consumption in cholesterol levels was observed while a significant decrease of inflammation was detected [105].

#### 3.3.2. Hypertension

Hypertension, in other words, high blood pressure, is another important risk factor for cardiovascular diseases, eventuating heart attacks and strokes [106]. High fat or salt consumption in the diet, obesity, cholesterol, and some hormone problems may induce hypertension. Hellström et al. investigated the blood pressure lowering activity of chokeberry in an in vivo study. They separated 40 rats into four groups which were fed with chokeberry polyphenolic extract, lyophilized chokeberry juice, and enalapril (ACE (Angiotensin-converting-enzyme) inhibitory drug) in addition to a control group. Daily consumption of 50 mg of chokeberry polyphenolic extract and chokeberry juice per kg of body weight caused a decrease in systolic blood pressure and diastolic blood pressure of rats. However, the authors also examined in vitro ACE inhibitory activity of polyphenolic extract and juice. It was pointed out that chokeberry juice exhibited an important ACE inhibitory activity while chokeberry polyphenol fractions performed lower activity. Authors remarked that the ACE inhibitory activity of chokeberry might be affected by non-phenolic compounds in the juice [107]. In a similar study, Sikora et al. observed weak in vitro ACE inhibitory activity for chokeberry extract. Despite this, in a clinical trial with metabolic syndrome patients, a 30% decrease in ACE enzyme activity was observed after two months of chokeberry extract supplementation [108]. According to a study performed by Yamane et al., the antihypertensive activity of the Aronia berry is related to the kidney renin-angiotensin system instead of inhibition of ACE in the lungs [109].

Studies on blueberries also reported antihypertensive activity. For instance, Wiseman et al. reported that feeding hypertensive stroke-prone rats with blueberries (3% of diet) for two weeks lowered ACE activity by 44%. After four weeks, ACE activity was prone to decrease equally in both blueberry consuming and non-consuming (control) groups of rats. However, at the end of six weeks, blueberry consumption led to a decrease by 12% in ACE activity [110]. Similar results were also obtained in clinical trials. For instance, consumption of blueberry by postmenopausal women with pre- and stage 1-hypertension resulted in a decrease in systolic blood pressure and diastolic blood pressure [111]. Other studies also indicated similar activities of decreasing blood pressure for haskap berry [112], cranberry [113], and strawberry [114].

### 3.4. Immune System

The immune system’s main responsibility is to resist infections in the body by the method of either innate or adaptive responses [115]. While innate responses have phagocytes, mediator releasing cells, and natural killer cells, the most notable cells of the adaptive immune system are lymphocytes named B and T cells. Briefly, B cells induce the formation of antibodies, while T cells terminate the infected units [116]. On the whole, the primary goal of the immune system could be simply described as detecting and destroying any attacks on the cells. The disorders of the immune system, both direct and indirect, are wide in variety and therefore it is extremely important to understand and enhance the responses of this system. The polyphenols in berries are known for having antiviral, antimicrobial, anti-inflammatory, and immunomodulatory properties on the immune system [117].

There have been a number of studies in the past decade that aim to highlight the specific effects and mechanisms of berries on the immune system and show their potential health benefits for humans. A study conducted in China investigated the immunomodulatory and anti-aging effects of goji berry, also known as wolfberry (*Lyciumbarbarum*), when combined with milk, namely lacto-wolfberry, over the course of 3 months [118]. The participants were aged between 65 and 70 years old and the study aimed to research if regular consumption of lacto-wolfberry would have enhancing effects after the participants were vaccinated for influenza. Their B cell and antibody productions were measured, and it was indicated that B cells were enhanced in terms of their activity rather than their numbers. Phytochemicals found in cranberries were investigated for their potential effects on influenza and the common cold, with a particular focus on the T cell gamma delta, γδ-T [119]. A total of 54 people consisting of 17 males and 37 females with varying ages and body indexes were observed over 10 weeks in March and May, taking into account the peak influenza and cold periods. The results showed that regular consumption of cranberry juice did not only lead to a decrease in symptoms, but also to app. 5 times increase of γδ-T cells and reduction of the release of inflammatory cytokine. A similar study was conducted with purple concord grape (*Vitis labrusca*) juice on 85 participants over 9 weeks [120]. The 26 men and 59 women in the study were elder adults between 50 and 75 years old with similar body mass indexes and did not consume any purple, red, or blue fruits during the study period. While the placebo group showed a decrease in serum antioxidant capacity, those consuming grape juice, with a total polyphenol content of 1894 mg GAE/L, did not experience any reduction in this sense. Furthermore, grape juice consumption was shown to have beneficial outcomes in regard to γδ-T cells and vitamin C levels. The immune related effects of chokeberry (*Aronia melanocarpa*) on both healthy mice and mice with type 1 diabetes were investigated due to the known antioxidant and anti-inflammatory characteristics of the fruit [121]. Analysis showed that the main anthocyanin, flavonoid, and phenolic acid compounds in the chokeberry extracts were cyanidin-3-galactoside (0.34 mg/g), quercetin-3-galactoside (0.31 mg/g), and chlorogenic acid (3.53 mg/g), respectively. It was seen that oral consumption of chokeberry induced both phagocytic cell and T cell activities, showing immunomodulatory significance as well as resulting in an escalation of blood sugar levels in mice with type 1 diabetes. These in vivo results were further supported by an in vitro model. Dendritic cells, frequently stated to be antigen-presenting units, are quite important as they lead to the activation of the adaptive immune system by interacting with T and B cells [122]. Ginseng berry extracts were shown to have a fairly high impact in terms of dendritic cell activation on mice, where similar positive outcomes were also observed in vitro [123]. Inflammatory bowel diseases (IBDs) are also noteworthy diseases, which bring about unfavorable living conditions [124]. The two main types are Crohn’s disease (CD) and ulcerative colitis (UC); the former primarily involves problems in the small and large intestine while the latter affects the large intestine and rectum [125]. Amongst environmental conditions, they are also caused by insufficiencies in the immune system [126]. There is no set cure for the treatment of IBD as of yet, therefore the treatment mainly involves easing the symptoms by controlling inflammation. Multiple in vivo and in vitro studies supported the positive impact of berries on IBD, relating it to the anti-inflammatory effects of the flavonoids they contain, with quercetin being a frequent mention [127]. A study conducted on the effects of maqui berry (*Aristotelia chilensis*) water extracts on mice with UC also supports this information [32]. Therapeutic, antioxidant, and anti-inflammatory impacts were observed in vivo as well as in the comparative in vitro model of the berry, due to its relatively high anthocyanin contents (0.17 ± 0.09 mg/g fraction) along with the total phenolics content (820.31 ± 9.37 mg/g). Goji berry was also shown to be potentially effective towards UC in mice through inflammation repression [128]. In addition, an extremely comprehensive review regarding the impact of diet on IBD details the effectiveness of different berries, amongst other fruits and legumes and states the potential protective abilities of combining the already-used drugs with anthocyanin-enriched supplements [129]. Although not the most traditional of berries, elderberries (*Sambucus nigra* L.) have also been used for years in the belief of their health benefits, especially against influenza and the common cold. Studies on mice showed that consumption of elderberries had negative effects on influenza A virus activity, which has been attributed to their high level of flavonoids [130]. The study states the total polyphenol and anthocyanin contents to be 42 mg/g and 6.6 mg/g, respectively. There are also a large number of studies in literature which show that the polyphenols abundant in berries, such as resveratrol, have enhancing properties in terms of the immune system [131]. In an extensive review regarding the health impact of berries on oral, esophageal, gastric, intestine, microbiome, and immune systems, it was stated that rather than the individual compounds that berries contain, such as quercetin, complete berry extracts seemed to provide more promise [39]. Given that there are various studies on the effects of different berries, and also an extreme variety of berries in nature, it can be deduced that further and more detailed studies could provide necessary information to help with the treatment of immune diseases.

### 3.5. Cancer

In addition to their well-known antioxidative and anti-inflammatory effects, berries containing bioactives such as polyphenolic anthocyanins have the ability to engage a multitude of signaling pathways with special molecular targets [132,133,134,135]. Furthermore, chemopreventive and therapeutic potential of berry extracts through apoptosis, cell proliferation, and angiogenesis motivate berries to be used as a tool for cancer treatments [63,132]. Antigenotoxicity and antimutagenicity of berries are one of the most popular fruit-based anticarcinogenic research topics and studies have been conducted in vitro, in vivo, or as clinical studies recently [136]. Many studies, which were extensively reviewed by Kristo et al., indicated the anticarcinogenic activities of berries based on anti-inflammation, angiogenesis inhibition, avoiding DNA damage, and decreasing the rates of apoptosis and proliferation of malignant cells [137]. In addition to these, the primary bioactive compounds that are present in many berry extracts are defined as epigallocatechin-3-gallate, quercetin, kaempferol, ellagic, chlorogenic, and gallic acids affected antioxidative, cell-cycle arrest, apoptosis, antiproliferative and anti-survival, anti-inflammation, anti-angiogenesis, metastasis inhibition, and cell adhesion/movement inhibition activities [137]. The majority of the studies on berries were focused on GI tract and breast cancers; however, other types of cancers have also been investigated [63,132,137]. For instance, in a systematic review, anticancer potential, as well as molecular action mechanism and toxicity of acai berry on various cancer types (esophageal cancer, urothelial cancer, melanoma, colon cancer, and Walker-256 tumor) were examined in detail based on clinical (pre and post) and in vivo studies [138].

The effects of berry bioactives on cancerous cell lines are the key actions correlated with the anticarcinogenic potentials. Black raspberry extract was found to diminish VEGF-induced cell migration, proliferation, and tube formation in human intestinal microvascular endothelial cells. TNF-α/IL-1β activation followed by VEGF stimulation was observed as an antiangiogenic effect of black raspberry extract which was mediated by Akt, MAPK, and JNK phosphorylation inhibitions [47]. Another similar significant inhibition of TNF-α (NF-ϰB) and (MCP)-1 was observed for ethanol induced gastric ulcer in rats when treated with black chokeberry hydro-alcoholic extract [57].

Anthocyanin-enriched Calafat berry extract was applied on gastric (AGC) and gallbladder (G415) human cancer cell lines at different concentrations (25–800 µg/mL). In vitro viability and migration capacities of gastric and gallbladder carcinoma human cell lines were observed to reduce significantly [139]. Blueberry and its leaf extracts were investigated for their anticarcinogenic as well as antioxidative properties with and without methyl jasmonate (MeJa) fortification on human gastric cancer cell lines (AGS) in vitro. A strong concurrent antioxidative activity, MeJa-fortified extracts significantly decreased cancer cell migration, gene expression for cancer-related proteins, and mainly the pathway of mitogen-activating protein kinase. However, no significant/important in vivo output was observed such as *helicobacter pylori* colonization, chemopreventive, or chemoprotective effects against gastric cancer [140].

Antigenotoxic activities of various berries on colorectal cancer types were discussed concerning the action mechanism, chemoprotective, and anticarcinogenic properties of berries aside from emphasizing the importance of gut microbiota covered by in vivo and in vitro studies [141]. Chilean blueberry and raspberry extracts were used to treat HT-29 and SW48 human colon cancer cell lines. Cell death ratios were observed as 80.1 and 72.5% for SW48 and HT-29 cell lines, respectively. When exposed to Chilean berry extracts, the cell growth inhibition was observed 90–100% when the extract was applied at the highest possible concentration (2000 µg/mL) [142]. In an in vitro study on human colonic mucosa cell cultures, oxidative stress-avoiding and antimutagenic properties of elderberry were assessed. Despite the colonic digestion loss of bioavailability, exposure to the 1 mg/mL freeze dried elderberry extract resulted in 22% reduction for intracellular ROS production, prevented the oxidative DNA damage at a level of 46%, and inhibited the oxidant-induced mutagenicity of *Salmonella typhimurium* TA102 strain at a ratio of 26% [143]. The effect of Maqui berry extract on human colon cancer cell lines (HT-29 and Caco-2) growth inhibition based on inflammatory mediators, which are COX-2 and NF-ϰB, were investigated. The results showed a significant decrease of growth of HT-29 and Caco-2 cell lines correlated with the inhibition of COX-2 and NF-ϰB due to the externally determined chemoprotective effect (with detailed antioxidative activity assays) of Maqui berry [144].

Non-small cell lung cancer cell lines were treated with a bioactive compound, 4β-hydroxywithanolide E (4HW), isolated from golden berry. It was indicated that 4HW treatment inhibited the tissue factor expression as well as procoagulant activity. Furthermore, the combination of 4HW with TNF-α triggered a synergistic cytotoxicity against targeted cell lines induced by caspase-dependent apoptosis. The action mechanism was briefly expressed as the reversing of TNF-α procoagulant activity together with enhancing its cytotoxicity by 4HW [145]. Degradation products of cyanidin-3-O-glucoside (C3G), the prominent anthocyanin in haskap berries, were obtained for their cytotoxic effects on hepatocellular carcinoma (HepG2) and breast cancer MDA-MB-231 cells. The C3G as well as the products, protocatechuic (PCA) and phloroglucinaldehyde (PGA), were applied on the targeted cell lines. C3G-enriched fraction of berry extract exhibited an inhibitory effect on HepG2 cell in a concentration-dependent manner for 48 h, while PCA provided a significant cytotoxic effect on both HepG2 and MDA-MB-231 cells [146]. Hydroethanolic extract of maqui berry was investigated for its inhibitory effect on human endometrial cancer cell line Ishikawa. The study based on cell cycle arrest or apoptosis induction demonstrated that the cell viability was inhibited while apoptosis increased with an EC50 of 427.3 µg/mL extract treatment along with lowered invasive capacity with unchanged migration of Ishikawa cells. Consequently, hydroethanolic extract of maqui berry exhibited promising antineoplastic properties for endometrial cancer [147]. In a study, Anthos berry bioactives were applied on drug sensitive (A2780) and drug resistant (A2780/CP70, OVCA432, and OVCA433) ovarian cancer cells. The significant inhibitory effect of Anthos bioactives was indicated when they were administered as milk-derived exosomes, in order to increase their oral bioavailability and stability. Eventually, exosomal Anthos decreased PgP (p-glycoprotein) level in a dose-dependent manner for both drug sensitive and drug resistant tumor xenografts significantly and was proposed as a promising antitumor agent coupled with an excellent nano drug carrier to manage the ovarian cancer [148]. The studies mainly conducted from a food science and nutrition perspective for the correlation in between the cancer tissue and berry phenolic compounds were non-existent, to the best of our knowledge, possibly requiring more complex analysis methodology of cancer studies. For that reason, only the effects of extracted/purified berry phenolics on cancerous cell lines (in vitro) were able to be covered in this section.

### 3.6. Nervous System

The nervous system, consisting of the central and the peripheral nervous systems, is responsible for identifying affecting environmental factors and transmitting responsive signals throughout the body. It is a remarkably complex system controlling the senses, physical movement, and consciousness [149]. Problems in the nervous system might cause a variety of conditions, such as anxiety and depression, and disorders, with some of the most well-known ones being Alzheimer’s disease, Parkinson’s disease, and Huntington’s disease [150]. Although these conditions and disorders have multiple causes, often in combination, research shows that the presence of ROS leading to oxidative stress is undeniably an affecting factor [151]. The leading organ is the brain, which is also a part of the central nervous system. The brain is quite susceptible to the adverse effects of ROS and unfortunately does not have the capacity to counteract the potential damage [40]. Multiple studies show the positive effects of berry consumption on reducing the stress caused by ROS on the nervous system.

#### 3.6.1. Alzheimer’s Disease

Alzheimer’s disease (AD) is one of the most well-known nervous system disorders. As the disease slowly deteriorates memory, its symptoms, beginning with basic forgetfulness, can lead to eventual loss of the ability to perform daily tasks, including speaking [152,153]. The causes are linked to various factors such as environmental exposures, stress, genetics, and infections, with the most dominant cause being age advancement [154]. While currently there is no definite cure for AD, there are various treatments aimed at alleviating the symptoms [155]. Multiple studies can be found in literature regarding the effect of berries on AD. Bilberry and blackcurrant were supplemented to mice to observe the inhibiting effects of AD [156]. The hindering effects of the berries on β-amyloid (Aβ) fibrils, which are an affecting factor of AD and lead to ROS generation, were studied. Although the berries exerted slightly different reductions in differing parameters, overall, it was found that both bilberry and blackcurrant exhibit a certain level of neuroprotective effect on mice with AD. In a study, which used the roundworm *Caenorhabditis elegans*, the effects of cranberries on AD were investigated [157]. Their results showed that the use of cranberries was able to decrease the Aβ toxicity and have promising preventative effects on AD, although its therapeutic result was not considered as promising. In another study lasting over a period of 16 weeks, mice were supplemented with blueberry extract to observe its effects on AD, particularly that of memory and learning advancements [158]. Their results showed that mice which consumed blueberry extract exhibited positive change in this sense when compared to the mice that were not treated. Additionally, a thorough study looking into the cognition improvement prospects of berries in terms of AD focused on mice treated with the polyphenolic extracts of blueberries and grapes. It was seen that even though Aβ fibrils were not hindered, the berry extract showed memory development [159].

#### 3.6.2. Parkinson’s Disease

Parkinson’s disease (PD) results in the gradual loss of control over both motor functions including tremor, rigidity, akinesia, and posture, and also non-motor functions such as cognitive and behavioral problems [160]. As with AD, there are numerous probable causes of the disease, ranging from lifestyle and heredity to age and surrounding conditions, with the most impacting factor being dopamine deficiency [161,162]. Dopamine is a neurotransmitter, widely associated with its impact on emotions [163]. Over the years, the treatment of PD symptoms has seen noteworthy advancement, with the standard being therapies focused on remedying dopamine deficiency via dopamine-based drugs, which among other effects, inhibit the generation of ROS [162]. Levodopa is the most widely known and used drug in the treatment of PD [164].

An extremely detailed study focusing on the long-term effects of flavonoid intake on PD was conducted [165]. The study consisted of looking into 5 different sources of flavonoids on 805 people over 20–22 years. Results showed that there was a considerable correlation between berry-sourced flavonoids and reduction of PD risk, where this risk decreased by 40% in men but was insignificant for women. Another study, specifically focused on mulberry fruit and its influence on neurotoxicity, showed similar positive results in both in vivo and in vitro PD models [166]. The observed behaviors, as well as brain tissues of mulberry treated mice indicated certain neuroprotective effects. In addition to the studies that focus on the effect of berries on PD, there are also various studies associated with the flavonoids, which are abundantly found in berry matrices. Various in vivo and in vitro studies focusing on fisetin, a flavonol abundant in strawberries, showed that it has the potential to treat PD symptoms [167]. Ellagic acid is a polyphenol present in berries which is also known to have ROS reducing effects. An in vivo study investigating the effect of ellagic acid on rats showed that ellagic acid was able to improve motor disturbances and showed potential in reducing free radical induced neural damage in PD [168].

#### 3.6.3. Huntington’s Disease

Huntington’s disease (HD) is a hereditary disease in 90% of cases, affecting cognition, behavior, and motor functions [169]. It is caused by the defective huntingtin gene on chromosome 4 leading to the coding of the huntingtin protein [170]. The symptoms of HD are similar to that of PD and include tremors and involuntary muscle contractions; however, the causes of HD are more precise. Although there is no cure at the moment for HD, there are various types of treatment for its symptoms [171].

In a very recent study, two types of raspberry extracts, namely *Rubus idaeus* and var Prestige, were investigated in terms of the BAC they contain [172]. Salidroside was the primary point of interest as it is a phenol known to be neuroprotective towards HD. *Saccharomyces cerevisiae* strains were used, and it was stated that this promising compound required further research to be used in medicine formulations.

#### 3.6.4. Other Nervous System-Related Symptoms

A study investigated the positive effects of blueberries on age-related attitude issues by comparing the behavior patterns of mice on high-fat and low-fat diets [173]. Using data from previous studies in literature, mice were put on high-fat diets, which caused behavioral problems due to ROS generation and inflammation. It was seen that blueberry supplementation on high-fat diet rats showed a significant improvement in their actions, with these rats exhibiting similar patterns to those fed low-fat diets. Although it was also stated that age and species of the mice could have differing impacts. Another study explored the anti-inflammatory effects of blackberries mainly composed of anthocyanins in their extracts on Wistar rats fed with a high-fat or a standard (control) diet, by the means of cytokine antibody assays [174]. Similar to the abovementioned study, the rats fed with a high-fat diet exhibited brain inflammation, which was significantly weakened by the berry extracts. A study was conducted focusing on potential neuroprotective effects of anthocyanins and acai berries extracts on *Caenorhabditis elegans* [175]. It was stated that in addition to the antioxidant and neuroprotective effects, the anthocyanin rich extracts were also able to mend the damage caused by Aβ. The chemotaxis of Aβ-expressing worms towards benzaldehyde are notably lowered; however, when treated with acai extracts, these worms showed a 402% increased chemosensory response. A different study on *Caenorhabditis elegans* showed that there was a likelihood of blueberries (*Vaccinium uliginosum*) and lingonberries (*Vaccinium vitis-idaea*) having advantages towards aging and neurodegenerative disorders [175]. An interesting study involving 35 beagle dogs between the ages of 8 and 15 explored how brain-related issues associated with aging would be influenced by berries [176]. The dogs were fed grape and blueberry extracts over 75 days, and the outcome showed potential benefits towards memory, caused by the positive effects of the berries towards oxidative stress. The beagles were subjected to a delayed non-matching to position test to evaluate their baseline memory performance and an enhanced cognitive performance was seen in relation to their baseline levels on dogs fed with the extracts. An in vitro comparative study of blackberry extracts examined their potential neuroprotective effects [177]. While the commercial blackberry did not exhibit any effects, the wild blackberries showed to be promising in this sense as they increased both mitochondrial transmembrane potential and cell membrane integrity. This difference was attributed to their varying polyphenol composition and antioxidant capacity with the *R. brigantinus* species having 60% higher antioxidant capacity than that of the commercial blackberry.

A brief summary of selected studies investigating the health effects of berry fruits is presented in Table 2.

## 4. Conclusions

The purpose of this review was to provide an overview of the recent studies investigating the health benefits of berry phenolics from the frame of food science and nutrition. As demonstrated from the studies covered in this paper, berry phenolics have protecting, supporting, alleviating, and curing potentials for many system diseases, including cardiovascular, immune, gastrointestinal, and nervous systems, when they are included in the routine daily diet. Furthermore, some studies state that they have a significant protective effect on cancer, which is a disease whose abundance is increasing due to today’s lifestyle as well as many other affecting factors. Along with this, it was also demonstrated quite repeatedly in literature that the positive effects of berries on health are related to the high levels of phenolic compounds they contain with varying types and contents differing for each berry species as well as other external factors such as climate or cultivation. Studies on the health promoting effects of berries are mostly animal based or in vitro clinical trials. Further research regarding how the outputs of animal studies could be interpreted/adapted for the human body is required in order to gain a more comprehensive understanding of the subject. Despite the positive effects observed in studies that were conducted so far, the mechanism of action for some diseases has not been fully explained and since berries have rich phenolic contents, they tend to interfere/act through more than one mechanism in some diseases. Nevertheless, further studies should be carried out to reveal the mechanisms of berry bioactives in order to better understand their promising health effects. Moreover, designing the studies to include different parameters and using a high number of subjects in the trials would help to provide the effect of treatment-supported consumption of berry fruits for individuals.

## Figures and Tables

**Table 1:** Commonly studied berries, their phenolic compounds, and total phenolic contents.

Berry Type	TPC	Expression/Unit	Commonly Found Phenolic Compounds	References
Blackberry	4016.43 ± 13.44	mg GAE/100 g DW	Cyanidin, ellagic acid, quercetin	[13,14,15,16,17]
Raspberry	735.03	mg GAE/100 g FW	Ellagic acid, quercetin, kaempferol, cyanidin	[13,17,18]
Blueberry	170.9–523.8	mg GAE/100 g FW	Chlorogenic acid, quercetin, myricetin, cyanidin	[18,19,20,21]
Chokeberry	1964–2782 mg	mg GAE/100 g DW	Quercetin, chlorogenic and neochlorogenic acids	[22,23,24,25]
Korean black raspberry	291.135	mg GAE/100 g FW	kaempferol, quercetin, ellagic acid	[22,26]
White grape	455–3113	mg GAE/100 g DW	Catechin, quercetin, kaempferol	[27,28,29]
Jostaberry	1593.92	mg GAE/100 g FW	Ellagic acid, quercetin, myricetin, kaempferol	[13,18]
Redcurrant	8.45	mg GAE/100 g FW	Quercetin, cyanidin, myricetin, kaempferol	[13,18,20]
Elderberry	3002	mg GAE/100 g DW	Cyanidin, Rutin, quercetin, gallic acid, gentisic acid	[30,31]
Maqui berry	4974 ± 57	mg GAE/100 g DW	Kaempferol, quercetin, myricetin, delphinidin, cyanidin	[32,33]

GAE: gallic acid equivalent; DW: dry weight; FW: fresh weight.

**Table 2:** Some selected studies related to the effect of berries on various health systems.

Berry/Extract	Disease/Disorder/System	Subject	Significant Effects	Reference
Lingonberry	Gastrointestinal system/oral cavity	In vivo (humans)	↓ viable counts, visible plaque index and probing bleeding	[44]
Black raspberry extract	Gastrointestinal system/esophagus	In vitro (microvascular endothelial cells)	Anti-inflammatory effect	[47]
Black chokeberry extract	Gastrointestinal system	In vivo (rats)	↓ gastric injury formation	[57]
Goji berry extract	Gastrointestinal system	In vitro	↑ the growth of probiotic bacteria	[64]
Golden berry	Metabolism/diabetes	In vivo (humans)	↓ blood glucose and ↑ insulin resistance	[72]
Mulberry extract	Metabolism/obesity	In vivo (hamsters)	↑ body weight ↑ lipolysis factor ↑ lipogenesis factor	[85]
Blueberry and mulberry	Metabolism/obesity	In vivo (mice)	↑ lipid accumulation	[87]
*Lonicera caerulea* berry	Cardiovascular system/cholesterol	In vivo (rats)	↓ total and LDL and ↑ HDL cholesterol in serum and liver	[90]
Aronia	Cardiovascular system/cholesterol	In vivo (humans)	↓ total and LDL cholesterol	[103]
Chokeberry	Cardiovascular system/hypertension	In vivo (rats)	↓ systolic and diastolic blood pressure	[107]
Blueberry	Cardiovascular system/hypertension	In vivo (rats)	↓ the ACE activity	[110]
Purple concord grape (*Vitis labrusca*)	Immune system	In vivo (humans)	↑ of γδ-T cells and ↓ of inflammatory cytokine release	[120]
Chokeberry	Immune system	In vivo (mice)	↑ phagocytic cell and T cell activities	[121]
Elderberry	Immune system	In vivo (mice)	↓ influenza A virus activity	[130]
Elderberry extract	Immune system	In vitro	↓ TNF-α and IFN-γ secretion	[178]
Calafat berry extract	Cancer	In vitro (gastric-AGC and gallbladder-G415 human cancer cell lines)	↓ viability and migration capacity	[139]
Maqui berry extract	Cancer	In vitro (human colon cancer cell lines (HT-29 and Caco-2))	↓ cell growth ratios	[144]
Golden berry extract	Cancer	In vitro (non-small cell lung cancer cell lines)	↓ tissue factor expression and procoagulant activity	[145]
Anthos berry extract	Cancer	In vitro (ovarian cancer cells)	↓ p-glycoprotein level	[148]
Bilberry	Cancer	In vivo and in vitro (oral squamous cell carcinoma cells-HSC-3 and zebrafish)	↓ viability, proliferation, migration and invasion of HSC-3 cells and tumor area in zebrafish	[179]
Blueberry extract	Cancer	In vitro (C6 rat glioma cell line)	↓ viability, proliferation, size of the colonies and cell migration	[180]
*Crataegus* berry extract	Cancer	In vitro (highly aggressive human glioblastoma U87MG cell line)	↓ proliferative and invasive potentials of glioblastoma cells	[181]
Andean berry	Cancer	In vitro (human SW480 colon adenocarcinoma cells)	↓ proliferative potential, ↑ late apoptosis stage	[182]
Blackberry extract	Cancer	In vitro (human stomach-AGS, colon-SW620, liver-HepG2 and skin-SK-Mel-28 cell lines)	↑ pro-apoptotic activity and early apoptosis stage	[183]
Cranberry (*Vaccinium macrocarpon*)	Alzheimer’s Disease	In vivo *(Caenorhabditis elegans)*	↔	[157]
Blueberry	Alzheimer’s Disease	In vivo (mice)	↑ memory and learning advancements	[158]
Goji berry powder	Alzheimer’s Disease	In vitro	↑ Cell viability ↓ Aβ production	[184]
Strawberry extract	Alzheimer’s Disease	In vivo (*Caenorhabditis elegans*)	↓ Aβ and ROS production	[183]
Blueberry extract	Alzheimer’s Disease	In vivo (mice)	↓ Aβ induced cytotoxicity ↑ Autophagy	[185]
Strawberry Alba extract	Alzheimer’s Disease	In vitro	↓ Oxidative DNA damage	[186]
Blueberry, Strawberry	Parkinson’s Disease	In vivo (human)	↓ the risk of disease	[165]
Mulberry	Parkinson’s Disease	In vivo (mice)	↔	[166]
Raspberry extracts (*Rubus idaeus* and var Prestige)	Huntington’s Disease	In vivo (*Saccharomyces cerevisiae*)	↔	[171]
Chilean berry (*Ugni* *Molinae*) extract	Huntington’s Disease	In vitro	↓ Abnormal protein aggregation	[187]
Blueberry (*Vaccinium ashei*)	Nervous System	In vivo (mice)	↓ recognition memory deficits	[172]
Blackberry	Nervous System	In vivo (Wistar rats)	↓ brain inflammation	[173]
Acai	Nervous System	In vivo (*Caenorhabditis elegans*)	↔ for neural activities	[174]
Blueberries (*Vaccinium uliginosum*) and lingonberries (*Vaccinium vitis-idaea*)	Nervous System	In vivo (*Caenorhabditis elegans*)	↔ against aging and neurodegenerative disorders	[175]
Grape and blueberry	Nervous System	In vivo (dogs)	↑ memory	[176]
Blackberry (*Rubus brigantinus, Rubus vagabundus*)	Nervous System	In vitro	↔ for neural activities	[177]

↔: Protective effect. ↑: Increased/induced. ↓: Decreased/reduced.

## References

[1] Magrone T., de Heredia F.P., Jirillo E., Morabito G., Marcos A., Serafini M. (2013). Functional foods and nutraceuticals as thera-peutic tools for the treatment of diet-related diseases. Can. J. Physiol. Pharmacol..

[2] Zhao Y. (2007). Berry Fruit: Value-Added Products for Health Promotion.

[3] Vendrame S., Del Bo’ C., Ciappellano S., Riso P., Klimis-Zacas D. (2016). Berry fruit consumption and metabolic syndrome. Antioxidants.

[4] Tsuda T. (2016). Recent Progress in Anti-Obesity and Anti-Diabetes Effect of Berries. Antioxidants.

[5] Stoner G.D., Wang L.-S., Zikri N., Chen T., Hecht S.S., Huang C., Sardo C., Lechner J.F. (2007). Cancer prevention with freeze-dried berries and berry components. Seminars in Cancer Biology.

[6] Forbes-Hernández T.Y. (2020). Berries polyphenols: Nano-delivery systems to improve their potential in cancer therapy. J. Berry Res..

[7] Joseph S.V., Edirisinghe I., Burton-Freeman B.M. (2014). Berries: Anti-inflammatory Effects in Humans. J. Agric. Food Chem..

[8] Anonym Berry Health Benefits Symposium. Berry Health Benefits Sym-Posium Offers a Unique Opportunity to Achieve Their Communication Goals. http://berryhealth.org/#:~:text=The.

[9] Skrovankova S., Sumczynski D., Mlcek J., Jurikova T., Sochor J. (2015). Bioactive Compounds and Antioxidant Activity in Different Types of Berries. Int. J. Mol. Sci..

[10] Nile S.H., Park S.W. (2014). Edible berries: Bioactive components and their effect on human health. Nutrition.

[11] Pandey K.B., Rizvi S.I. (2009). Plant Polyphenols as Dietary Antioxidants in Human Health and Disease. Oxid. Med. Cell. Longev..

[12] Cianciosi D., Simal-Gándara J., Forbes-Hernández T.Y. (2019). The importance of berries in the human diet. Med. J. Nutr. Metab..

[13] Okatan V. (2020). Antioxidant properties and phenolic profile of the most widely appreciated cultivated berry species: A compara-tive study. Folia Hortic..

[14] Zafra-Rojas Q., Cruz-Cansino N., Delgadillo-Ramírez A., Alanís-García E., Añorve-Morga J., Lira A.Q., Castañeda-Ovando A., Ramírez-Moreno E. (2018). Organic Acids, Antioxidants, and Dietary Fiber of Mexican Blackberry (Rubus fruticosus) Residues cv. Tupy. J. Food Qual..

[15] Kafkas E., Koşar M., Türemiş N., Başer K.H.C. (2006). Analysis of sugars, organic acids and vitamin C contents of blackberry gen-otypes from Turkey. Food Chem..

[16] Oszmiański J., Nowicka P., Teleszko M., Wojdyło A., Cebulak T., Oklejewicz K. (2015). Analysis of Phenolic Compounds and Antioxidant Activity in Wild Blackberry Fruits. Int. J. Mol. Sci..

[17] Lugasi A., Hóvári J., Kádár G., Denes F. (2011). Phenolics in raspberry, blackberry and currant cultivars grown in Hungary. Acta Aliment..

[18] Kasım R., Şanlıbaba P., Kasım M.U. (2017). The Antioxidant Effects of Berry Fruits. International Congress on Medicinal and Aromatic Plants.

[19] Carvalho M.J., Gouveia C.S., Vieira A.C., Pereira A.C., Carvalho M., Marques J.C. (2017). Nutritional and phytochemical com-position of Vaccinium padifolium sm wild berries and radical scavenging activity. J. Food Sci..

[20] Gavrilova V., Kajdžanoska M., Gjamovski V., Stefova M. (2011). Separation, Characterization and Quantification of Phenolic Compounds in Blueberries and Red and Black Currants by HPLC−DAD−ESI-MSn. J. Agric. Food Chem..

[21] Kim J.G., Kim H.L., Kim S.J., Park K.-S. (2013). Fruit quality, anthocyanin and total phenolic contents, and antioxidant activities of 45 blueberry cultivars grown in Suwon, Korea. J. Zhejiang Univ. Sci. B.

[22] Suh D.H., Jung E.S., Lee G.M., Lee C.H. (2018). Distinguishing six edible berries based on metabolic pathway and bioactivity cor-relations by non-targeted metabolite profiling. Front. Plant Sci..

[23] Sidor A., Gramza-michałowska A. (2019). Black Chokeberry Aronia Melanocarpa L.—A Qualitative Composition, Phenolic Profile and Antioxidant Potential. Molecules.

[24] Kapci B., Neradová E., Čížková H., Voldřich M., Rajchl A., Capanoglu E. (2013). Investigating the antioxidant potential of chokeberry (Aronia melanocarpa) products. J. Food Nutr. Res..

[25] Ćujić N., Šavikin K., Janković T., Pljevljakušić D., Zdunić G., Ibrić S. (2016). Optimization of polyphenols extraction from dried chokeberry using maceration as traditional technique. Food Chem..

[26] Yang J.W., Choi I.S. (2016). Comparison of the phenolic composition and antioxidant activity of Korean black raspberry, Bokbunja, (Rubus coreanusMiquel) with those of six other berries. CyTA-J. Food.

[27] Mullen W., Marks S.C., Crozier A. (2007). Evaluation of Phenolic Compounds in Commercial Fruit Juices and Fruit Drinks. J. Agric. Food Chem..

[28] José Jara-Palacios M., Hernanz D., Escudero-Gilete M.L., Heredia F.J. (2014). Antioxidant potential of white grape pomaces: Phe-nolic composition and antioxidant capacity measured by spectrophotometric and cyclic voltammetry methods. Food Res. Int..

[29] Castillo-Muñoz N., Gómez-Alonso S., García-Romero E., Hermosín-Gutiérrez I. (2010). Flavonol profiles of Vitis vinifera white grape cultivars. J. Food Compos. Anal..

[30] Domínguez R., Zhang L., Rocchetti G., Lucini L., Pateiro M., Munekata P.E.S., Lorenzo J.M. (2020). Elderberry (*Sambucus nigra* L.) as potential source of antioxidants. Characterization, optimization of extraction parameters and bioactive properties. Food Chem..

[31] Duymuş H.G., Göger F., Başer K.H.C. (2014). In vitro antioxidant properties and anthocyanin compositions of elderberry extracts. Food Chem..

[32] Zhou G., Chen L., Sun Q., Mo Q.-G., Sun W.-C., Wang Y.-W. (2019). Maqui berry exhibited therapeutic effects against DSS-induced ulcerative colitis in C57BL/6 mice. Food Funct..

[33] Genskowsky E., Puente L.A., Pérez-Álvarez J.A., Fernández-López J., Muñoz L.A., Viuda-Martos M. (2016). Determination of polyphenolic profile, antioxidant activity and antibacterial properties of maqui [Aristotelia chilensis (Molina) Stuntz] a Chilean blackberry. J. Sci. Food Agric..

[34] Xiao F., Xu T., Lu B., Liu R. (2020). Guidelines for antioxidant assays for food components. Food Front..

[35] Venskutonis P.R., Galanakis C. (2020). Valorization of Fruit Processing By-Products.

[36] Zorzi M., Gai F., Medana C., Aigotti R., Morello S., Peiretti P.G. (2020). Bioactive Compounds and Antioxidant Capacity of Small Berries. Foods.

[37] Kelly E., Vyas P., Weber J.T. (2017). Biochemical Properties and Neuroprotective Effects of Compounds in Various Species of Berries. Molecules.

[38] Mazzoni L., Perez-Lopez P., Giampieri F., Alvarez-Suarez J.M., Gasparrini M., Forbes-Hernandez T.Y., Quiles J.L., Mezzetti B., Battino M. (2016). The genetic aspects of berries: From field to health. J. Sci. Food Agric..

[39] Govers C., Kasikci M.B., Van Der Sluis A.A., Mes J.J. (2018). Review of the health effects of berries and their phytochemicals on the digestive and immune systems. Nutr. Rev..

[40] Foito A., McDougall G.J., Stewart D. (2018). Evidence for Health Benefits of Berries. Annu. Plant Rev. Online.

[41] Bhattacharyya A., Chattopadhyay R., Mitra S., Crowe S.E. (2014). Oxidative stress: An essential factor in the pathogenesis of gas-trointestinal mucosal diseases. Physiol. Rev..

[42] Sangiovanni E., Fumagalli M., Dell’Agli M. (2017). Berries: Gastrointestinal Protection against Oxidative Stress and Inflammation.

[43] Philip N., Walsh L.J. (2019). Cranberry Polyphenols: Natural Weapons against Dental Caries. Dent. J..

[44] Pärnänen P., Nikula-Ijäs P., Sorsa T. (2019). Antimicrobial and anti-inflammatory lingonberry mouthwash—A clinical pilot study in the oral cavity. Microorganisms.

[45] Ben Lagha A., Howell A., Grenier D. (2020). Highbush blueberry proanthocyanidins alleviate Porphyromonas gingivalis-induced deleterious effects on oral mucosal cells. Anaerobe.

[46] Kranz S., Guellmar A., Olschowsky P., Tonndorf-Martini S., Heyder M., Pfister W., Reise M., Sigusch B. (2020). Antimicrobial Effect of Natural Berry Juices on Common Oral Pathogenic Bacteria. Antibiotics.

[47] Medda R., Lyros O., Schmidt J.L., Jovanovic N., Nie L., Link B.J., Otterson M.F., Stoner G.D., Shaker R., Rafiee P. (2015). Anti inflammatory and anti angiogenic effect of black raspberry extract on human esophageal and intestinal microvascular endo-thelial cells. Microvasc. Res..

[48] Jabri M.-A., Tounsi H., Rtibi K., Marzouki L., Sakly M., Sebai H. (2016). Ameliorative and antioxidant effects of myrtle berry seed (Myrtus communis) extract during reflux-induced esophagitis in rats. Pharm. Biol..

[49] Jabri M.-A., Marzouki L., Sebai H. (2018). Ethnobotanical, phytochemical and therapeutic effects of Myrtus communis L. berries seeds on gastrointestinal tract diseases: A review. Arch. Physiol. Biochem..

[50] Jabri M.-A., Rtibi K., Tounsi H., Hosni K., Marzouki L., Sakly M., Sebai H. (2017). Fatty acid composition and mechanisms of the protective effects of myrtle berry seed aqueous extract in alcohol-induced peptic ulcer in rat. Can. J. Physiol. Pharmacol..

[51] Giampieri F., Cianciosi D., Forbes-Hernández T.Y. (2020). Myrtle (*Myrtus communis* L.) berries, seeds, leaves, and essential oils: New undiscovered sources of natural compounds with promising health benefits. Food Front..

[52] Sangiovanni E., Vrhovsek U., Rossoni G., Colombo E., Brunelli C., Brembati L., Trivulzio S., Gasperotti M., Mattivi F., Bosisio E. (2013). Ellagitannins from Rubus Berries for the Control of Gastric Inflammation: In Vitro and In Vivo Studies. PLoS ONE.

[53] Si W.Z., Hu X.X., Shen C., Luo M.X., Xuan Y.H., Fu H.J., Xing G., Zhang H.F., Zhang J., Liu Z. (2020). Protective Effect of Schisandra chinensis extract against ethanol-induced gastric ulcers in mice by promoting anti-inflammatory and mucosal defense mechanisms. Rev. Bras. Farmacogn..

[54] Jabri M.-A., Rtibi K., Sakly M., Marzouki L., Sebai H. (2016). Role of gastrointestinal motility inhibition and antioxidant properties of myrtle berries (*Myrtus communis* L.) juice in diarrhea treatment. Biomed. Pharmacother..

[55] Nesello L.A.N., Beleza M.L.M.L., Mariot M., Mariano L.N.B., de Souza P., Campos A., Cechinel-Filho V., Andrade S.F., Da Silva L.M. (2017). Gastroprotective Value of Berries: Evidences from Methanolic Extracts of Morus nigra and Rubus niveus Fruits. Gastroenterol. Res. Pract..

[56] Cury B.J., Boeing T., Somensi L.B., Mariano L.N.B., de Andrade S.F., Breviglieri E., Klein-Junior L.C., de Souza P., da Silva L.M. (2020). Açaí berries (Euterpe oleracea Mart.) dried extract improves ethanol-induced ulcer in rats. J. Pharm. Pharmacol..

[57] Paulrayer A., Adithan A., Lee J.H., Moon K.H., Kim D.G., Im S.Y., Kang C.-W., Kim N.S., Kim J.-H. (2017). Aronia melanocarpa (Black Chokeberry) Reduces Ethanol-Induced Gastric Damage via Regulation of HSP-70, NF-κB, and MCP-1 Signaling. Int. J. Mol. Sci..

[58] Matsumoto M., Hra H., Chiji H., Kasai T. (2004). Gastroprotective effect of red pigments in black chokeberry fruit (Aroniamelano-carpa Elliot) on acute gastric hemorrhagic lesions in rats. J. Agric. Food Chem..

[59] Valcheva-Kuzmanova S., Denev P., Eftimov M., Georgieva A., Kuzmanova V., Kuzmanov A., Kuzmanov K., Tzaneva M. (2019). Protective effects of Aronia melanocarpa juices either alone or combined with extracts from Rosa canina or Alchemilla vul-garis in a rat model of indomethacin-induced gastric ulcers. Food Chem. Toxicol..

[60] Valcheva-Kuzmanova S., Kuzmanov A., Kuzmanova V., Tzaneva M. (2018). Aronia melanocarpa fruit juice ameliorates the symptoms of inflammatory bowel disease in TNBS-induced colitis in rats. Food Chem. Toxicol..

[61] Hsieh S.Y., Lian Y.Z., Lin I.H., Yang Y.C., Tinkov A.A., Skalny A.V., Chao J.C.J. (2021). Combined Lycium babarum polysac-charides and C-phycocyanin increase gastric Bifidobacterium relative abundance and protect against gastric ulcer caused by aspirin in rats. Nutr. Metab..

[62] Agulló V., González-Trujano M.E., Hernandez-Leon A., Estrada-Camarena E., Pellicer F., García-Viguera C. (2021). Antino-ciceptive effects of maqui-berry (Aristotelia chilensis (Mol.) Stuntz). Int. J. Food Sci. Nutr..

[63] Lavefve L., Howard L.R., Carbonero F. (2020). Berry polyphenols metabolism and impact on human gut microbiota and health. Food Funct..

[64] Skenderidis P., Mitsagga C., Lampakis D., Petrotos K., Giavasis I. (2019). The Effect of Encapsulated Powder of Goji Berry (*Lycium barbarum*) on Growth and Survival of Probiotic Bacteria. Microorganisms.

[65] Nagamani S., Sahoo R., Muneeswaran G., Narahari Sastry G. (2019). Data Science Driven Drug Repurposing for Metabolic Dis-orders. In Silico Drug Design.

[66] (2016). Global Report on Diabetes.

[67] Krentz A.J., Patel M.B., Bailey C.J. (2008). New drugs for type 2 diabetes mellitus: What is their place in therapy?. Drugs.

[68] Sénéchal M., Slaght J., Bharti N., Bouchard D.R. (2014). Independent and combined effect of diet and exercise in adults with pre-diabetes. Diabetes Metab. Syndr. Obes. Targets Ther..

[69] Ullah H., De Filippis A., Santarcangelo C., Daglia M. (2020). Epigenetic regulation by polyphenols in diabetes and related compli-cations. Med. J. Nutr. Metab..

[70] Edirisinghe I., Burton-Freeman B. (2016). Anti-diabetic actions of Berry polyphenols—Review on proposed mechanisms of action. J. Berry Res..

[71] Jeon Y.-D., Kang S.-H., Moon K.-H., Lee J.-H., Kim D.-G., Kim W., Kim J.-S., Ahn B.-Y., Jin J.-S. (2018). The Effect of Aronia Berry on Type 1 Diabetes In Vivo and In Vitro. J. Med. Food.

[72] Kinasih L.S., Djamiatun K., Al-Baarri A.N. (2020). Golden berry (Physalis peruviana) juice for reduction of blood glucose and ame-lioration of insulin resistance in diabetic rats. J. Gizi Dan Pangan.

[73] Sharma A., Kim J.W., Ku S.-K., Choi J.-S., Lee H.-J. (2019). Anti-diabetic effects of blue honeyberry on high-fed-diet-induced type II diabetic mouse. Nutr. Res. Pr..

[74] Martineau L.C., Couture A., Spoor D., Benhaddou-Andaloussi A., Harris C., Meddah B., Leduc C., Burt A., Vuong T., Le P.M. (2006). Anti-diabetic properties of the Canadian lowbush blueberry Vaccinium angustifolium Ait. Phytomedicine.

[75] Whyte A.R., Rahman S., Bell L., Edirisinghe I., Krikorian R., Williams C.M., Burton-Freeman B. (2020). Improved metabolic function and cognitive performance in middle-aged adults following a single dose of wild blueberry. Eur. J. Nutr..

[76] Smith T.J., Karl J.P., Wilson M.A., Whitney C.C., Barrett A., Farhadi N.F., Chen C.-Y.O., Montain S.J. (2019). Glycaemic regulation, appetite and ex vivo oxidative stress in young adults following consumption of high-carbohydrate cereal bars fortified with polyphenol-rich berries. Br. J. Nutr..

[77] Paquette M., Larqué A.S.M., Weisnagel S.J., Desjardins Y., Marois J., Pilon G., Dudonné S., Marette A., Jacques H. (2017). Strawberry and cranberry polyphenols improve insulin sensitivity in insulin-resistant, non-diabetic adults: A parallel, double-blind, controlled and randomised clinical trial. Br. J. Nutr..

[78] Ji Y., Liu D., Jin Y., Zhao J., Zhao J., Li H., Li L., Zhang H., Wang H. (2021). In vitro and in vivo inhibitory effect of anthocya-nin-rich bilberry extract on α-glucosidase and α-amylase. LWT.

[79] Boath A.S., Stewart D., McDougall G.J. (2012). Berry components inhibit α-glucosidase in vitro: Synergies between acarbose and polyphenols from black currant and rowanberry. Food Chem..

[80] Liu S., Yu J., Guo S., Fang H., Chang X. (2020). Inhibition of pancreatic α-amylase by Lonicera caerulea berry polyphenols in vitro and their potential as hyperglycemic agents. LWT.

[81] Muceniece R., Klavins L., Kviesis J., Jekabsons K., Rembergs R., Saleniece K., Dzirkale Z., Saulite L., Riekstina U., Klavins M. (2019). Antioxidative, hypoglycaemic and hepatoprotective properties of five Vaccinium spp. berry pomace extracts. J. Berry Res..

[82] Porras-Mija I., Chirinos R., García-Ríos D., Aguilar-Galvez A., Huaman-Alvino C., Pedreschi R., Campos D. (2020). Physico-chemical characterization, metabolomic profile and in vitro antioxidant, antihypertensive, antiobesity and antidiabetic properties of Andean elderberry (Sambucus nigra subsp. peruviana). J. Berry Res..

[83] Wright S.M., Aronne L.J. (2012). Causes of obesity Abdominal Imaging. Imaging.

[84] McDougall G.J., Stewart D. (2005). The inhibitory effects of berry polyphenols on digestive enzymes. BioFactors.

[85] Peng C.-H., Liu L.-K., Chuang C.-M., Chyau C.-C., Wang C.-J. (2011). Mulberry Water Extracts Possess an Anti-obesity Effect and Ability To Inhibit Hepatic Lipogenesis and Promote Lipolysis. J. Agric. Food Chem..

[86] Saponaro C., Gaggini M., Carli F., Gastaldelli A. (2015). The Subtle Balance between Lipolysis and Lipogenesis: A Critical Point in The Subtle Balance between Lipolysis and Lipogenesis: A Critical Point in Metabolic Homeostasis. Nutrients.

[87] Wu T., Tang Q., Gao Z., Yu Z., Song H., Zheng X., Chen W. (2013). Blueberry and Mulberry Juice Prevent Obesity Development in C57BL/6 Mice. PLoS ONE.

[88] Wu T., Gao Y., Guo X., Zhang M., Gong L. (2018). Blackberry and Blueberry Anthocyanin Supplementation Counteract High-Fat-Diet-Induced Obesity by Alleviating Oxidative Stress and Inflammation and Accelerating Energy Expenditure. Oxidative Med. Cell. Longev..

[89] Aranaz P., Romo-Hualde A., Zabala M., Navarro-Herrera D., de Galarreta M.R., Gil A.G., Martinez J.A., Milagro F.I., González-Navarro C.J. (2017). Freeze-dried strawberry and blueberry attenuates diet-induced obesity and insulin resistance in rats by inhibiting adipogenesis and lipogenesis. Food Funct..

[90] Liu J., Hao W., He Z., Kwek E., Zhu H., Ma N., Ma K.Y., Chen Z.Y. (2021). Blueberry and cranberry anthocyanin extracts re-duce bodyweight and modulate gut microbiota in C57BL/6 J mice fed with a high-fat diet. Eur. J. Nutr..

[91] Kowalska K., Olejnik A., Rychlik J., Grajek W. (2014). Cranberries (Oxycoccus quadripetalus) inhibit adipogenesis and lipogenesis in 3T3-L1 cells. Food Chem..

[92] Kowalska K., Olejnik A., Rychlik J., Grajek W. (2015). Cranberries (Oxycoccus quadripetalus) inhibit lipid metabolism and modulate leptin and adiponectin secretion in 3T3-L1 adipocytes. Food Chem..

[93] Chae H.S., You B.H., Choi J., Chin Y.W., Kim H., Choi H.S., Choi Y.H. (2019). Ginseng berry extract enhances metformin efficacy against obesity and hepatic steatosis in mice fed high-fat diet through increase of metformin uptake in liver. J. Funct. Foods.

[94] WHO Cardiovascular Diseases (CVDs). https://www.who.int/en/news-room/fact-sheets/detail/cardiovascular-diseases-.

[95] (2007). Prevention of Cardiovascular Disease Guidelines for Assessment and Management of Cardiovascular Risk.

[96] Hawkins J., Hires C., Baker C., Keenan L., Bush M. (2021). Daily supplementation with aronia melanocarpa (chokeberry) reduces blood pressure and cholesterol: A meta analysis of controlled clinical trials. J. Diet. Suppl..

[97] Blood Cholesterol|NHLBI, NIH. https://www.nhlbi.nih.gov/health-topics/blood-cholesterol.

[98] Liu S., Wu Z., Guo S., Meng X., Chang X. (2018). Polyphenol-rich extract from wild Lonicera caerulea berry reduces cholesterol accumulation by mediating the expression of hepatic miR-33 and miR-122, HMGCR, and CYP7A1 in rats. J. Funct. Foods.

[99] Liu S., You L., Zhao Y., Chang X. (2018). Wild Lonicera caerulea berry polyphenol extract reduces cholesterol accumulation and enhances antioxidant capacity in vitro and in vivo. Food Res. Int..

[100] Kim B., Ku C.S., Pham T.X., Park Y., Martin D.A., Xie L., Taheri R., Lee J., Bolling B.W. (2013). Aronia melanocarpa (chokeberry) polyphenol-rich extract improves antioxidant function and reduces total plasma cholesterol in apolipoprotein E knockout mice. Nutr. Res..

[101] Furlan C.P.B., Valle S.C., Maróstica M.R., Östman E., Björck I., Tovar J. (2019). Effect of bilberries, lingonberries and cinnamon on cardiometabolic risk-associated markers following a hypercaloric-hyperlipidic breakfast. J. Funct. Foods.

[102] Nilsson A., Salo I., Plaza M., Björck I. (2017). Effects of a mixed berry beverage on cognitive functions and cardiometabolic risk markers; A randomized cross-over study in healthy older adults. PLoS ONE.

[103] Xie L., Vance T., Kim B., Gil Lee S., Caceres C., Wang Y., Hubert P.A., Lee J.-Y., Chun O.K., Bolling B.W. (2017). Aronia berry polyphenol consumption reduces plasma total and low-density lipoprotein cholesterol in former smokers without lowering biomarkers of inflammation and oxidative stress: A randomized controlled trial. Nutr. Res..

[104] Naruszewicz M., Łaniewska I., Millo B., Dłużniewski M. (2007). Combination therapy of statin with flavonoids rich extract from chokeberry fruits enhanced reduction in cardiovascular risk markers in patients after myocardial infraction (MI). Atherosclerosis.

[105] Loo B.M., Erlund I., Koli R., Puukka P., Hellström J., Wähälä K., Mattila P., Jula A. (2016). Consumption of chokeberry (Aronia mitschurinii) products modestly lowered blood pressure and reduced low-grade inflammation in patients with mildly elevated blood pressure. Nutr. Res..

[106] (2013). A Global Brief on Hypertension|A Global Brief on Hyper Tension.

[107] Hellström J.K., Shikov A.N., Makarova M.N., Pihlanto A.M., Pozharitskaya O.N., Ryhänen E.-L., Kivijärvi P., Makarov V.G., Mattila P.H. (2010). Blood pressure-lowering properties of chokeberry (Aronia mitchurinii, var. Viking). J. Funct. Foods.

[108] Sikora J., Broncel M., Mikiciuk-Olasik E. (2014). Aronia melanocarpa elliot reduces the activity of angiotensin I-converting enzyme-In vitro and EX vivo studies. Oxid. Med. Cell. Longev..

[109] Yamane T., Kozuka M., Imai M., Yamamoto Y., Ohkubo I., Sakamoto T., Nakagaki T., Nakano Y. (2017). Reduction of blood pressure by aronia berries through inhibition of angiotensin-converting enzyme activity in the spontaneously hypertensive rat kidney. Funct. Foods Heal. Dis..

[110] Wiseman W., Egan J.M., Slemmer J.E., Shaughnessy K.S., Ballem K., Gottschall-Pass K.T., Sweeney M.I. (2011). Feeding blueberry diets inhibits angiotensin ii-converting enzyme (ACE) activity in spontaneously hypertensive stroke-prone rats. Can. J. Physiol. Pharmacol..

[111] Johnson S.A., Figueroa A., Navaei N., Wong A., Kalfon R., Ormsbee L.T., Feresin R.G., Elam M.L., Hooshmand S., Payton M.E. (2015). Daily Blueberry Consumption Improves Blood Pressure and Arterial Stiffness in Postmenopausal Women with Pre- and Stage 1-Hypertension: A Randomized, Double-Blind, Placebo-Controlled Clinical Trial. J. Acad. Nutr. Diet..

[112] Bell L., Williams C.M. (2019). A pilot dose–response study of the acute effects of haskap berry extract (Lonicera caerulea L.) on cogni-tion, mood, and blood pressure in older adults. Eur. J. Nutr..

[113] Novotny J.A., Baer D.J., Khoo C., Gebauer S.K., Charron C.S. (2015). Cranberry juice consumption lowers markers of cardiomet-abolic risk, including blood pressure and circulating c-reactive protein, triglyceride, and glucose concentrations in adults. J. Nutr..

[114] Feresin R.G., Johnson S.A., Pourafshar S., Campbell J.C., Jaime S.J., Navaei N., Elam M.L., Akhavan N.S., Alvarez-Alvarado S., Tenenbaum G. (2017). Impact of daily strawberry consumption on blood pressure and arterial stiffness in pre- and stage 1-hypertensive postmenopausal women: A randomized controlled trial. Food Funct..

[115] Delves P.J., Roitt I.M. (2000). The Immune System. N. Engl. J. Med..

[116] Alberts B., Johnson A., Lewis J., Raff M., Roberts K., Walter P., Alberts B., Johnson A., Lewis J., Raff M., Roberts K., Walter P. (2002). The Adaptive Immune System. Molecular Biology of the Cell.

[117] Gramza-Michałowska A., Sidor A., Kulczyński B. (2017). Berries as a potential anti-influenza factor—A review. J. Funct. Foods.

[118] Vidal K., Bucheli P., Gao Q., Moulin J., Shen L.-S., Wang J., Blum S., Benyacoub J. (2012). Immunomodulatory Effects of Dietary Supplementation with a Milk-Based Wolfberry Formulation in Healthy Elderly: A Randomized, Double-Blind, Placebo-Controlled Trial. Rejuvenation Res..

[119] Nantz M.P., Rowe C.A., Muller C., Creasy R., Colee J., Khoo C., Percival S.S. (2013). Consumption of cranberry polyphenols enhances human γδ-T cell proliferation and reduces the number of symptoms associated with colds and influenza: A randomized, placebo-controlled intervention study. Nutr. J..

[120] Rowe C.A., Nantz M.P., Nieves C., West R.L., Percival S.S. (2011). Regular Consumption of Concord Grape Juice Benefits Human Immunity. J. Med. Food.

[121] Gajić D., Saksida T., Koprivica I., Vujicic M., Despotovic S., Savikin K., Jankovic T., Stojanovic I. (2020). Chokeberry (Aronia melanocarpa) fruit extract modulates immune response in vivo and in vitro. J. Funct. Foods.

[122] Vroman H., van den Blink B., Kool M. (2015). Mode of dendritic cell activation: The decisive hand in Th2/Th17 cell differentiation. Implications in asthma severity?. Immunobiology.

[123] Zhang W., Cho S.-Y., Xiang G., Min K.-J., Yu Q., Jin J.-O. (2015). Ginseng Berry Extract Promotes Maturation of Mouse Dendritic Cells. PLoS ONE.

[124] Schirbel A., Reichert A., Roll S., Baumgart D.C., Büning C., Wittig B., Wiedenmann B., Dignass A., Sturm A. (2010). Impact of pain on health-related quality of life in patients with inflammatory bowel disease. World J. Gastroenterol..

[125] Vezza T., Rodríguez-Nogales A., Algieri F., Utrilla M.P., Rodriguez-Cabezas M.E., Galvez J. (2016). Flavonoids in Inflammatory Bowel Disease: A Review. Nutrients.

[126] Baumgart D.C., Carding S. (2007). Inflammatory bowel disease: Cause and immunobiology. Lancet.

[127] Pérez-Cano F.J., Castell M. (2016). Flavonoids, Inflammation and Immune System. Nutrients.

[128] Kang Y., Xue Y., Du M., Zhu M.-J. (2017). Preventive effects of Goji berry on dextran-sulfate-sodium-induced colitis in mice. J. Nutr. Biochem..

[129] Farzaei M.H., Elsenduny F., Momtaz S., Parvizi F., Iranpanah A., Tewari D., Naseri R., Abdolghaffari A.H., Rezaei N. (2018). An update on dietary consideration in inflammatory bowel disease: Anthocyanins and more. Expert Rev. Gastroenterol. Hepatol..

[130] Kinoshita E., Hayashi K., Katayama H., Hayashi T., Obata A. (2012). Anti-influenza virus effects of elderberry juice and its fractions. Biosci. Biotechnol. Biochem..

[131] Yuan J., Lu L., Zhang Z., Zhang S. (2012). Dietary Intake of Resveratrol Enhances the Adaptive Immunity of Aged Rats. Rejuvenation Res..

[132] Bishayee A., Haskell Y., Do C., Siveen K.S., Mohandas N., Sethi G., Stoner G.D. (2015). Potential Benefits of Edible Berries in the Management of Aerodigestive and Gastrointestinal Tract Cancers: Preclinical and Clinical Evidence. Crit. Rev. Food Sci. Nutr..

[133] De Cedrón M.G., Wagner S., Reguero M., Menéndez-Rey A., De Molina A.R. (2020). Miracle Berry as a Potential Supplement in the Control of Metabolic Risk Factors in Cancer. Antioxidants.

[134] Qamar M., Akhtar S., Ismail T., Sestili P., Tawab A., Ahmed N. (2020). Anticancer and anti-inflammatory perspectives of Paki-stan’s indigenous berry Grewia asiatica Linn (Phalsa). J. Berry Res..

[135] Giampieri F., Gasparrini M., Forbes-Hernandez T.Y., Mazzoni L., Capocasa F., Sabbadini S., Alvarez-Suarez J.M., Afrin S., Rosati C., Pandolfini T. (2018). Overexpression of the Anthocyanidin Synthase Gene in Strawberry Enhances Antioxidant Capacity and Cytotoxic Effects on Human Hepatic Cancer Cells. J. Agric. Food Chem..

[136] Izquierdo-Vega J.A., Morales-González J.A., Sánchez-Gutiérrez M., Betanzos-Cabrera G., Sosa-Delgado S.M., Sumaya-Martínez M.T., Morales-González Á., Paniagua-Pérez R., Madrigal-Bujaidar E., Madrigal-Santillán E. (2017). Evidence of Some Natural Products with Antigenotoxic Effects. Part 1: Fruits and Polysaccharides. Nutrients.

[137] Kristo A., Klimis-Zacas D., Sikalidis A. (2016). Protective Role of Dietary Berries in Cancer. Antioxidants.

[138] Alessandra-Perini J., Rodrigues-Baptista K.C., Machado D.E., Nasciutti L.E., Perini J.A. (2018). Anticancer potential, molecular mechanisms and toxicity of Euterpe oleracea extract (açaí): A systematic review. PLoS ONE.

[139] Calderón-Reyes C., Pezoa R.S., Leal P., Ribera-Fonseca A., Cáceres C., Riquelme I., Zambrano T., Peña D., Alberdi M., Reyes-Díaz M. (2020). Anthocyanin-Rich Extracts of Calafate (Berberis microphylla G. Forst.) Fruits Decrease In Vitro Viability and Migration of Human Gastric and Gallbladder Cancer Cell Lines. J. Soil Sci. Plant Nutr..

[140] Ribera-Fonseca A., Jiménez D., Leal P., Riquelme I., Roa J.C., Alberdi M., Peek R.M., Reyes-Díaz M. (2020). The anti-proliferative and anti-invasive effect of leaf extracts of blueberry plants treated with methyl jasmonate on human gastric cancer in vitro is related to their antioxidant properties. Antioxidants.

[141] Brown E., Latimer C., Allsopp P., Ternan N., McMullan G., McDougall G.J., Stewart D., Crozier A., Rowland I., Gill C.I.R. (2014). In Vitro and in Vivo Models of Colorectal Cancer: Antigenotoxic Activity of Berries. J. Agric. Food Chem..

[142] Flis S., Jastrzebski Z., Namiesnik J., Arancibia-Avila P., Toledo F., Leontowicz H., Leontowicz M., Suhaj M., Trakhtenberg S., Gorinstein S. (2012). Evaluation of inhibition of cancer cell proliferation in vitro with different berries and correlation with their antioxidant levels by advanced analytical methods. J. Pharm. Biomed. Anal..

[143] Olejnik A., Olkowicz M., Kowalska K., Rychlik J., Dembczyński R., Myszka K., Juzwa W., Białas W., Moyer M.P. (2016). Gas-trointestinal digested Sambucus nigra L. fruit extract protects in vitro cultured human colon cells against oxidative stress. Food Chem..

[144] Céspedes-Acuña C.L., Xiao J., Wei Z.-J., Chen L., Bastias J.M., Avila J.G., Alarcon-Enos J., Werner-Navarrete E., Kubo I. (2018). Antioxidant and anti-inflammatory effects of extracts from Maqui berry Aristotelia chilensis in human colon cancer cells. J. Berry Res..

[145] Hsieh K.-Y., Tsai J.-Y., Lin Y.-H., Chang F.-R., Wang H.-C., Wu C.-C. (2021). Golden berry 4β-hydroxywithanolide E prevents tumor necrosis factor α-induced procoagulant activity with enhanced cytotoxicity against human lung cancer cells. Sci. Rep..

[146] Pace E., Jiang Y., Clemens A., Crossman T., Rupasinghe H.V. (2018). Impact of Thermal Degradation of Cyanidin-3-O-Glucoside of Haskap Berry on Cytotoxicity of Hepatocellular Carcinoma HepG2 and Breast Cancer MDA-MB-231 Cells. Antioxidants.

[147] Mena J., Elgueta E., Espinola-Gonzales F., Cardenas H., Orihuela P.A. (2021). Hydroethanolic Extracts of the Aristotelia Chilensis (Maqui) Berry Reduces Cellular Viability and Invasiveness in the Endometrial Cancer Cell Line Ishikawa. Integr. Cancer Ther..

[148] Aqil F., Jeyabalan J., Agrawal A.K., Kyakulaga A.-H., Munagala R., Parker L., Gupta R.C. (2017). Exosomal delivery of berry anthocyanidins for the management of ovarian cancer. Food Funct..

[149] Snyder J.M., Hagan C.E., Bolon B., Keene C.D. (2018). Nervous System. Comparative Anatomy and Histology.

[150] Salim S. (2017). Oxidative Stress and the Central Nervous System. J. Pharmacol. Exp. Ther..

[151] Umeno A., Biju V., Yoshida Y. (2017). In vivo ROS production and use of oxidative stress-derived biomarkers to detect the onset of diseases such as Alzheimer’s disease, Parkinson’s disease, and diabetes. Free. Radic. Res..

[152] Lyketsos C.G., Carrillo M.C., Ryan J.M., Khachaturian A.S., Trzepacz P., Amatniek J., Cedarbaum J., Brashear R., Miller D.S. (2011). Neuropsychiatric symptoms in Alzheimer’s disease. Alzheimer’s Dement..

[153] Cera M.L., Ortiz K.Z., Bertolucci P.H.F., Minett T.S.C. (2013). Speech and orofacial apraxias in Alzheimer’s disease. Int. Psychogeriatr..

[154] Armstrong R.A. (2013). What causes Alzheimer’s disease?. Folia Neuropathol..

[155] Fish P.V., Steadman D., Bayle E.D., Whiting P. (2019). New approaches for the treatment of Alzheimer’s disease. Bioorganic Med. Chem. Lett..

[156] Vepsäläinen S., Koivisto H., Pekkarinen E., Mäkinen P., Dobson G., McDougall G.J., Stewart D., Haapasalo A., Karjalainen R.O., Tanila H. (2013). Anthocyanin-enriched bilberry and blackcurrant extracts modulate amyloid precursor protein processing and alleviate behavioral abnormalities in the APP/PS1 mouse model of Alzheimer’s disease. J. Nutr. Biochem..

[157] Guo H., Dong Y.-Q., Ye B.-P. (2016). Cranberry extract supplementation exerts preventive effects through alleviating Aβ toxicity in Caenorhabditis elegans model of Alzheimer’s disease. Chin. J. Nat. Med..

[158] Tan L., Yang H., Pang W., Li H., Liu W., Sun S., Song N., Zhang W., Jiang Y. (2017). Investigation on the Role of BDNF in the Benefits of Blueberry Extracts for the Improvement of Learning and Memory in Alzheimer’s Disease Mouse Model. J. Alzheimer’s Dis..

[159] Dal-Pan A., Dudonné S., Bourassa P., Bourdoulous M., Tremblay C., Desjardins Y., Calon F. (2016). Cognitive-Enhancing Effects of a Polyphenols-Rich Extract from Fruits without Changes in Neuropathology in an Animal Model of Alzheimer’s Disease. J. Alzheimer’s Dis..

[160] Politis M., Wu K., Molloy S., Bain P.G., Chaudhuri K.R., Piccini P. (2010). Parkinson’s disease symptoms: The patient’s perspective. Mov. Disord..

[161] Beitz J.M. (2014). Parkinson’s disease: A review. Front. Biosci..

[162] Emamzadeh F.N., Surguchov A. (2018). Parkinson’s Disease: Biomarkers, Treatment, and Risk Factors. Front. Neurosci..

[163] Ko J.H., Strafella A.P. (2012). Dopaminergic neurotransmission in the human brain: New lessons from perturbation and imaging. Neuroscientist.

[164] Smith Y., Wichmann T., Factor S.A., DeLong M.R. (2011). Parkinson’s Disease Therapeutics: New Developments and Challenges Since the Introduction of Levodopa. Neuropsychopharmacol..

[165] Gao X., Cassidy A., Schwarzschild M.A., Rimm E.B., Ascherio A. (2012). Habitual intake of dietary flavonoids and risk of Par-kinson disease. Neurology.

[166] Kim H.G., Ju M.S., Shim J.S., Kim M.C., Lee S.H., Huh Y., Kim S.Y., Oh M.S. (2010). Mulberry fruit protects dopaminergic neu-rons in toxin-induced Parkinson’s disease models. Br. J. Nutr..

[167] Maher P. (2017). Protective effects of fisetin and other berry flavonoids in Parkinson’s disease. Food Funct..

[168] Farbood Y., Sarkaki A., Dolatshahi M., Mansouri S.M.T., Khodadadi A. (2015). Ellagic Acid Protects the Brain Against 6-Hydroxydopamine Induced Neuroinflammation in a Rat Model of Parkinson’s Disease. Basic Clin. Neurosci. J..

[169] Dayalu P., Albin R.L. (2015). Huntington Disease: Pathogenesis and Treatment. Neurol. Clin..

[170] Frank S. (2014). Treatment of Huntington’s Disease. Neurotherapeutics.

[171] Kallscheuer N., Menezes R., Foito A., Da Silva M.H., Braga A., Dekker W., Sevillano D.M., Rosado-Ramos R., Jardim C., Oliveira J. (2019). Identification and microbial production of the raspberry phenol salidroside that is active against hun-tington’s disease. Plant Physiol..

[172] Carey A.N., Gomes S.M., Shukitt-Hale B. (2014). Blueberry Supplementation Improves Memory in Middle-Aged Mice Fed a High-Fat Diet. J. Agric. Food Chem..

[173] Meireles M., Marques C., Norberto S., Fernandes I., Mateus N., Rendeiro C., Spencer J.P., Faria A., Calhau C. (2015). The impact of chronic blackberry intake on the neuroinflammatory status of rats fed a standard or high-fat diet. J. Nutr. Biochem..

[174] Peixoto H., Roxo M., Krstin S., Wang X., Wink M. (2016). Anthocyanin-rich extract of Acai (Euterpe precatoria Mart.) mediates neuroprotective activities in Caenorhabditis elegans. J. Funct. Foods.

[175] Touray F., Maulik M., Scerbak C., Vayndorf E., Ito A.B., Taylor B. (2018). Alaskan berry treatments maintain C. elegans motility and sarcomere integrity with age. FASEB J..

[176] Fragua V., Lepoudère A., Leray V., Baron C., Araujo J.A., Nguyen P., Milgram N.W. (2017). Effects of dietary supplementation with a mixed blueberry and grape extract on working memory in aged beagle dogs. J. Nutr. Sci..

[177] Tavares L., Figueira I., McDougall G.J., Vieira H.L.A., Stewart D., Alves P.M., Ferreira R.B., Santos C.N. (2013). Neuroprotec-tive effects of digested polyphenols from wild blackberry species. Eur. J. Nutr..

[178] Schön C., Mödinger Y., Krüger F., Doebis C., Pischel I., Bonnländer B. (2021). A new high-quality elderberry plant extract exerts antiviral and immunomodulatory effects in vitro and ex vivo. Food Agric. Immunol..

[179] Mauramo M., Onali T., Wahbi W., Vasara J., Lampinen A., Mauramo E., Kivimäki A., Martens S., Häggman H., Sutinen M. (2021). Bilberry (*Vaccinium myrtillus* L.) Powder Has Anticarcinogenic Effects on Oral Carcinoma In Vitro and In Vivo. Antioxidants.

[180] da Silveira L.M., Pedra N.S., Bona N.P., Spohr L., da Silva dos Santos F., Saraiva J.T., Alvez F.L., Meine B.D.M., Spanevello R.M., Stefanello F.M. (2021). Selective in vitro anticancer effect of blueberry extract (*Vaccinium virgatum*) against C6 rat glioma: Exploring their redox status. Metab. Brain Dis..

[181] Żurek N., Karatsai O., Rędowicz M.J., Kapusta I.T. (2021). Polyphenolic Compounds of Crataegus Berry, Leaf, and Flower Ex-tracts Affect Viability and Invasive Potential of Human Glioblastoma Cells. Molecules.

[182] Arango-Varela S.S., Luzardo-Ocampo I., Reyes-Dieck C., Yahia E.M., Maldonado-Celis M.E. (2021). Antiproliferative potential of Andean Berry (Vaccinium meridionale Swartz) juice in combination with Aspirin in human SW480 colon adenocarcinoma cells. J. Food Biochem..

[183] Navarro-Hortal M.D., Romero-Márquez J.M., Esteban-Muñoz A., Sánchez-González C., Rivas-García L., Llopis J., Quiles J.L. (2022). Strawberry (Fragaria× ananassa cv. Romina) methanolic extract attenuates Alzheimer’s beta amyloid production and oxidative stress by SKN-1/NRF and DAF-16/FOXO mediated mechanisms in C. elegans. Food Chem..

[184] Binosha Fernando W.M., Dong K., Durham R., Stockmann R., Jayasena V. (2021). Effect of goji berry on the formation of extra-cellular senile plaques of Alzheimer’s disease. Nutr. Healthy Aging..

[185] Li H., Zheng T., Lian F., Xu T., Yin W., Jiang Y. (2022). Anthocyanin-rich blueberry extracts and anthocyanin metabolite proto-catechuic acid promote autophagy-lysosomal pathway and alleviate neurons damage in in vivo and in vitro models of Alz-heimer’s disease. Nutrition.

[186] Živković L., Bajić V., Čabarkapa-Pirković A., Dekanski D., Forbes-Hernández T.Y., Zlatković-Švenda M., Spremo-Potparević B. (2021). Strawberry (Fragaria ananassa duch.) Alba extract attenuates DNA damage in lymphocytes of patients with Alzheimer’s disease. J. Food Biochem..

[187] Pérez-Arancibia R., Ordoñez J.L., Rivas A., Pihán P., Sagredo A., Ahumada U., Barriga A., Seguel I., Cárdenas C., Vidal R.L. (2021). A phenolic-rich extract from Ugni molinae berries reduces abnormal protein aggregation in a cellular model of Huntington’s disease. PLoS ONE.

